# Identification of telomerase RNAs in species of the *Yarrowia* clade provides insights into the co-evolution of telomerase, telomeric repeats and telomere-binding proteins

**DOI:** 10.1038/s41598-019-49628-6

**Published:** 2019-09-16

**Authors:** Filip Červenák, Katarína Juríková, Hugo Devillers, Binyamin Kaffe, Areej Khatib, Erin Bonnell, Martina Sopkovičová, Raymund J. Wellinger, Jozef Nosek, Yehuda Tzfati, Cécile Neuvéglise, Ľubomír Tomáška

**Affiliations:** 1 Departments of Genetics and Biochemistry, Comenius University in Bratislava, Faculty of Natural Sciences, Ilkovičova 6, Mlynská dolina, 84215 Bratislava, Slovakia; 2 Micalis Institute, INRA, AgroParisTech, Université Paris-Saclay, 78350 Jouy-en-Josas, France; 3 Department of Genetics, The Silberman Institute of Life Sciences, The Hebrew University of Jerusalem, Safra Campus, Jerusalem, 91904 Israel; 4 Department of Microbiology and Infectiology, RNA Group, Faculty of Medicine and Health Sciences, Université de Sherbrooke, Sherbrooke, Québec J1E 4K8 Canada

**Keywords:** Evolutionary biology, Telomeres

## Abstract

Telomeric repeats in fungi of the subphylum Saccharomycotina exhibit great inter- and intra-species variability in length and sequence. Such variations challenged telomeric DNA-binding proteins that co-evolved to maintain their functions at telomeres. Here, we compare the extent of co-variations in telomeric repeats, encoded in the telomerase RNAs (TERs), and the repeat-binding proteins from 13 species belonging to the *Yarrowia* clade. We identified putative TER loci, analyzed their sequence and secondary structure conservation, and predicted functional elements. Moreover, *in vivo* complementation assays with mutant TERs showed the functional importance of four novel TER substructures. The TER-derived telomeric repeat unit of all species, except for one, is 10 bp long and can be represented as 5′-TTNNNNAGGG-3′, with repeat sequence variations occuring primarily outside the vertebrate telomeric motif 5′-TTAGGG-3′. All species possess a homologue of the *Yarrowia lipolytica* Tay1 protein, *Yl*Tay1p. *In vitro*, *Yl*Tay1p displays comparable DNA-binding affinity to all repeat variants, suggesting a conserved role among these species. Taken together, these results add significant insights into the co-evolution of TERs, telomeric repeats and telomere-binding proteins in yeasts.

## Introduction

Telomeres are dynamic and complex nucleoprotein structures located at the ends of linear chromosomes. Their principal function is to solve both the end-replication and the end-protection problems, while also contributing to the regulation of gene expression, chromosome movement and localization^1–6^. In most eukaryotic organisms, telomeres of nuclear chromosomes are composed of short DNA tandem repeats arranged in a double-stranded array and terminating in 3′ single-stranded (usually G-rich) overhang. The prevalent mechanism of telomere maintenance is based on a specialized reverse transcriptase called telomerase. The catalytic subunit of telomerase (TERT, Est2 in budding yeast) employs an RNA molecule (TER) as a scaffold, an anchor to the telomere and a template for the elongation of the 3′ overhang^7^. Telomeric repeats are bound by specific DNA-binding proteins, which, along with their interaction partners, form the shelterin complex^6,8–11^. These proteins protect telomeric DNA against recombination and undue DNA repair throughout most of the cell cycle, but relinquish telomeres to telomerase and DNA polymerases during S-phase^12,13^.

In order to ensure the dynamic switching between the inaccesible and extensible states of telomeres, when the 3′ overhang can be associated with telomerase and elongated, telomeric repeats have to satisfy specific criteria: telomeric proteins need to bind them strongly enough to secure the correct assembly of shelterin, but they also need to be able to transiently dissociate from telomeric DNA to allow its spatio-temporal accessibility to replication and transcription machineries^14–16^.

Given their essential roles in maintaining linear chromosomes, it is not surprising that the fundamental features of telomeres are conserved. On the other hand, there are several notable variations in how the conserved concepts are achieved. Perhaps the most dramatic deviation is represented by *Drosophila* and related species, where the short telomeric repeats are replaced by long complex retrotransposons^17^. Another example of variability in the mechanisms of telomere maintenance is represented by chromosomal termini of ascomycetous yeasts. Their telomeric sequences are rapidly evolving in both primary sequence and length^18–20^ and, with the exception of fission yeasts, their telomeric nucleoprotein complex differs significantly from the typical vertebrate shelterin. The double-stranded regions of telomeres in these species are bound by a variety of distantly related Myb-domain containing proteins^21^ and the CST complex regulates the maintenance of both G- and C-rich telomeric strands^22^. The reason for this rapidly divergent evolution of telomeres in yeasts is unknown. However, it is clear that the changes in sequences of telomeric repeats, derived from altered TER templates and degenerate template use, are accompanied by co-evolution of telomere-binding proteins, whose structures are adapting to the rapidly evolving cognate DNA^21^. In some phylogenetic branches, especially those comprising species that contain heterogenous telomeric repeats, this selection pressure resulted in the emergence of proteins exhibiting flexibility in DNA-binding specificity (such as Rap1 from *Saccharomyces cerevisiae*^20^ or Taz1 from *Schizosacharomyces pombe*^23^). Ascomycetous yeasts thus provide an ideal opportunity to study the co-evolution of TERs, telomeric repeats and the DNA-binding proteins that recognize them.

TERs from several yeast species, including *S*. *cerevisiae*, *S*. *pombe* and *Kluyveromyces lactis*, were thoroughly studied in the past, revealing the mechanism by which the sequence of the template domain of TER specifies the sequence of the telomeric repeats^24–29^. The extremely high degree of variability in length and sequence of TERs, even in closely related species, was later attributed to the fact that parts of TER may serve as a flexible scaffold for the assembly of protein complexes which bind only specific small parts of the RNA molecule^30^. The interactions between TER and a number of regulatory proteins were reported to modulate the activity, processivity and stability of the telomerase complex, affecting the overall telomere length and possibly also the sequence of telomeric repeats. For example, in *S*. *cerevisiae*, the variability of telomeric repeats is caused by low nucleotide incorporation processivity of telomerase^29,31–34^. Moreover, the important roles of specific RNA chaperones such as TCAB1 and assembly regulators including Lar7 and Pof8 in regulation of telomerase activity were described recently^35–37^. Comparative and functional analyses of yeast TERs also revealed several domains responsible for the assembly of the catalytic core of telomerase (pseudoknot, three-way junction (TWJ)), defining the telomeric repeat (template domain, template boundary element) and interaction with additional protein regulators (Ku-binding domain, Est1-binding domain, Sm site, Pop6, Pop7-binding/P3-like domain)^18,38–44^. In addition, several features of TER sequence facilitate spliceosomal cleavage, a special type of post-transcriptional processing discovered in *S*. *pombe*^45^. In this process, the TER precursor RNA is processed by the spliceosome, but undergoes only the first trans-esterification reaction of splicing, which releases the first exon as a mature TER^45^. The required sequence features differ among the yeast species where spliceosomal cleavage was later identified as a mode of TER processing, including the *Schizosaccharomyces* genus, *Aspergillus* and *Neurospora crassa*^18,46–48^.

Some functional domains are present in all TERs, although their sequence is not completely conserved, while others were described only in specific lineages (e.g. the snoRNA typical H/ACA box is conserved in vertebrate TERs, but not present in yeast TERs). In such cases, the presence or absence of a specific feature in TER often depends on the presence and activity of its interaction partner, so the co-evolution of TERs and their regulatory proteins takes place side by side with the co-evolution of telomeric repeats and telomere-binding proteins^49,50^. Hence, detailed analyses of conserved domains of TERs from phylogenetically distant species, such as those of filamentous fungi^46,51^, as well as the identification of novel features with potential to reveal previously unknown TER-protein interactions, are instrumental for better understanding of the evolution of yeast telomeric repeats and ultimately the entire telomere-protecting system.

Tracing back the actual evolutionary steps which led to the present variability of yeast telomeric repeats is difficult because most of these sequences are too divergent for simple alignment and the identification of conserved positions is complicated by their variable length. However, the telomeric repeat of *Yarrowia lipolytica* (5′-TTagtcAGGG-3′), which belongs to one of the basal phylogenetical lineages of Saccharomycotina, is relatively short, includes the canonical vertebrate motif 5′-TTAGGG-3′ and is bound both *in vitro* and *in vivo* by Tay1p^52,53^. Tay1 protein exhibits high affinity binding to both mammalian and *Y*. *lipolytica* telomeric repeats with stronger preference for the mammalian type^54^. Although it lacks a putative dimerization region, it contains two Myb domains exhibiting higher similarity to the Myb domains of human TRF1 and TRF2 than to Myb domains of other yeast telomere-binding proteins such as Taz1 or Rap1^53^. Interestingly, Rap1p from several yeast species also possess 2 Myb domains, which are able to form a stable complex with the DNA^55^, but the overall structural and sequential similarity between Rap1p and Tay1p as well as their respective Myb domains is limited^21^. Homologues of Tay1p in other species are either absent or play roles at nontelomeric loci^23,56,57^ reflecting a major deviation of their telomeric repeats from the 5′-TTAGGG-3′ sequence. *Y*. *lipolytica* may therefore serve as a model of the ancestral tipping point, where the canonical type of telomere was converted to its divergent derivatives present in other ascomycetous yeasts.

To investigate this transition in more detail, we focused on analyses of TERs, telomeric repeats and telomere-binding proteins from 13 species belonging to the *Yarrowia* clade^58^, i.e. twelve species of the genus *Yarrowia* and *Candida hispaniensis*, which was used as an outgroup. These species exhibit a pronounced sequence divergence, with *Y*. *galli* and *Y*. *deformans* being the closest species to *Y*. *lipolytica* with 87–89% similarity in average between orthologs^59^. *Y*. *alimentaria* and *Y*. *phangngensis* appear to be the most divergent and dynamic species, as exemplified by the evolution of the lipase gene family^60^. The comparison of telomeric repeat sequences between individual species enabled us to identify conserved positions within a repeat required for the binding by Tay1p homologs that seem to retain their telomeric functions. We identified putative genomic loci for TER in all species and showed that a ∆*ter* mutant of *Y*. *lipolytica* undergoes rapid (yet reversible) shortening of telomeres. Comparative analysis of TER sequences resulted in the identification of conserved as well as novel structural elements. These elements were subjected to functional analysis to test their involvement in proper functioning of telomerase *in vivo*. Our results illustrate how investigation of telomeres in *Yarrowia* clade species may be instrumental in understanding the paths that eventually resulted in an unprecedented diversification of telomeres in yeasts.

## Results

### Identification and characterization of TER loci in the *Yarrowia* clade species

**Figure 1:**
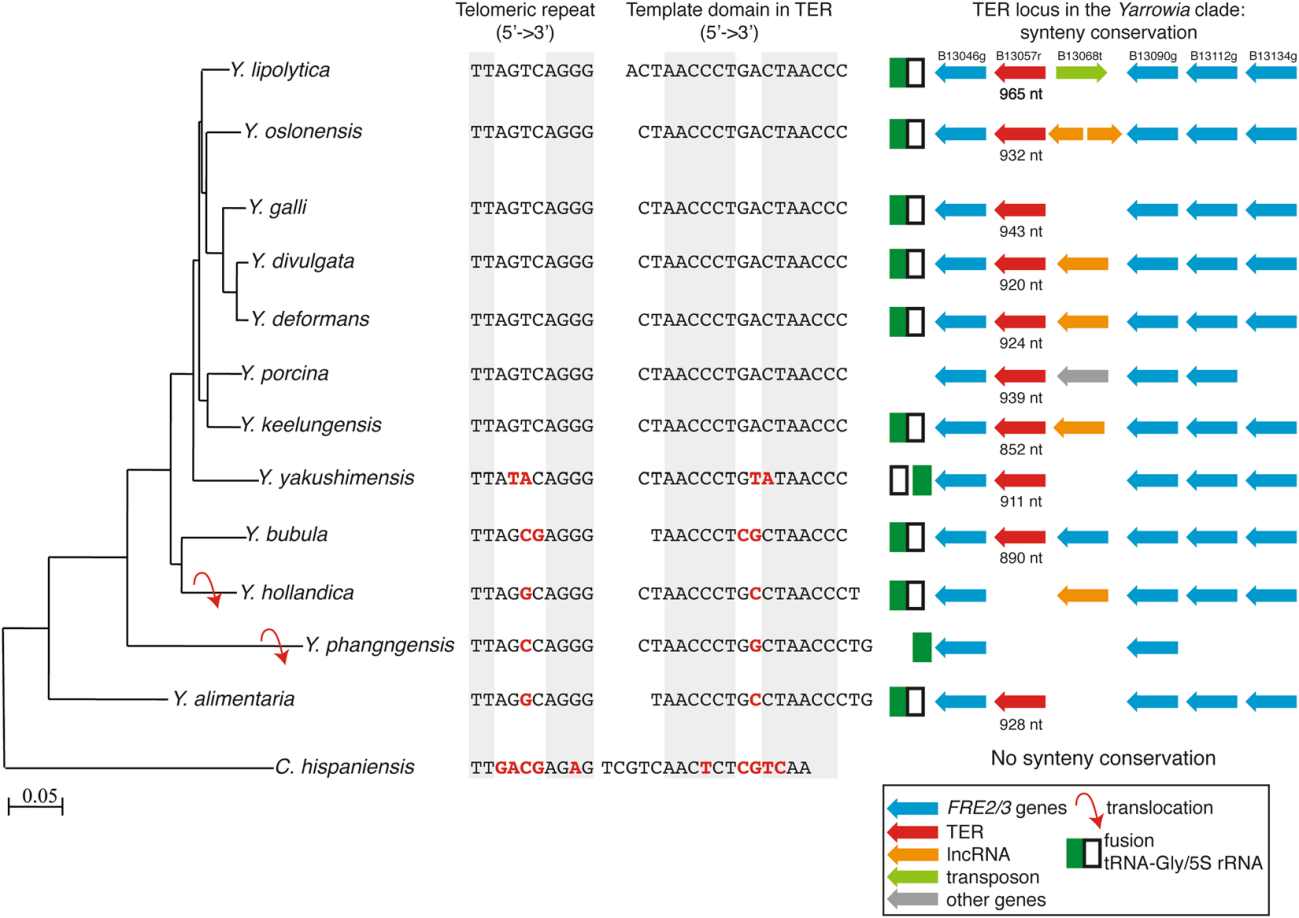
Comparison of the sequences of telomeric repeats and template domains of TERs in the *Yarrowia* clade species. Bases in red indicate substitutions in the telomeric repeat and the template domain of TER, respectively, compared with *Y*. *lipolytica* TER. Sequences in grey boxes represent the vertebrate telomeric motif (Telomeric repeat column) or the template sequence required for its synthesis (Template domain in TER column).

To identify the TER locus of *Y*. *lipolytica*, we first performed a BlastN search of its genome using the sequence of 1.5 telomeric repeat (5′-GTTAGTCAGGGTTAG-3′) as a query. Using this approach we identified a single intergenic locus (TER locus) on chromosome B that is actively transcribed. Although based on our RNA-seq analysis (see also below), the longest possible TER transcripts would be 1400–1500 nt long, ribo-depleted RNA-seq data showed that most reads mapped to this area end at ~950 nt downstream from the 5′ end of TER (Supplementary Fig. S1). The *Y*. *lipolytica* TER locus lies between two open reading frames encoding homologs of *S*. *cerevisiae FRE2* (YALI0B13046g) and *FRE3* (YALI0B13090g) genes (Fig. 1). In the reference genome of strain E150, the TER locus is flanked on one side by a retrotransposon that is absent in strain H222-S4 used for functional analyses. Similar search criteria as for *Y*. *lipolytica* were used to identify the putative TER loci in other species and the final candidates were chosen with respect to synteny within the TER locus containing region in *Y*. *lipolytica*. The synteny was retained in most of the species with the exceptions of *Y*. *hollandica* and *Y*. *phangngensis*, where the TER locus was transferred into another part of the genome due to two independent translocational events (Supplementary Fig. S2). In the case of *C*. *hispaniensis*, further changes in the genome structure altered the positions of both TER locus and neighbouring genes, resulting in the loss of synteny in this species. The coordinates of the TER in each species were determined with RNA-seq data (data not shown).

### Evolution of telomeric repeats in the species belonging to the *Yarrowia* clade

The sequences of telomeric repeats of 12 species belonging to the *Yarrowia* clade were determined by searching the ends of the scaffolds for short repeats resembling the telomeric sequence of *Y*. *lipolytica* or identifying these sequences in predicted TERs (see below) and deducing the exact sequence of the repeat from the conserved template region. In all 13 species (including *Y*. *lipolytica*) the telomeric repeat unit is composed of 10 nucleotides. Except for *C*. *hispaniensis*, these repeats are represented by a sequence 5′-TTNNNNAGGG-3′, where a canonical (vertebrate-type) telomeric repeat is interrupted by an insertion of four nucleotides (Fig. 1). This indicates that 5′-TT—AGGG-3′ sequences are less prone to substitutions than the spacer region, probably reflecting the binding preferences of telomere-binding proteins in the *Yarrowia* clade species (see below). More dramatic changes within the telomeric repeat occurred in *C*. *hispaniensis*, where four substitutions within the spacer region were accompanied by one change (G-to-A) in the canonical part of the repeat (Fig. 1). This reflects a relatively distant relationship of *C*. *hispaniensis* to the other species of this group.

**Figure 2:**
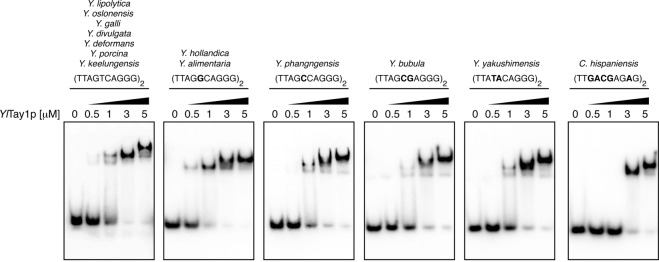
Effect of the substitutions in telomeric repeats on the affinity of *Yl*Tay1p. EMSA experiments used purified *Yl*Tay1p at the indicated concentrations and end-labelled double-stranded DNA probes (with the shown sequences corresponding to the telomeric repeat of the listed species).

Next, we asked whether the changes in telomeric repeats are reflected by the amino acid sequences of Myb domains of Tay1, the major telomere-binding protein in *Y*. *lipolytica*. Comparison of the Myb domains of Tay1p homologs from the *Yarrowia* clade species revealed that in the 7 species containing the *Y*. *lipolytica*-type telomeric repeat (5′-TTAGTCAGGG-3′) the Myb domains are with one exception identical (Supplementary Fig. S3). In *Y*. *porcina* there is one (Myb1) and two (Myb2) substitutions whose effect on affinity of the protein to telomeres was not tested. Importantly, in three out of five species with a single or double substitutions in the spacer region of the repeat (*Y*. *bubula*, *Y*. *hollandica* and *Y*. *phangngensis*), both Myb domains are identical to those of *Yl*Tay1 protein. Interestingly, even in *C*. *hispaniensis*, where 5 out of 10 nucleotides within the repeat sequence are different when compared with *Y*. *lipolytica*, the Myb domains of Tay1p homologue experienced only 4 (Myb1) and 2 (Myb2) amino acid substitutions that may be associated with changes in their specificity toward the telomeric repeats. Yet, the conservation of Myb domains of Tay1p homologues in the species with a variant version of telomeric repeat implies that the protein either tolerates the changes in the spacer part of the telomeric sequence, or it was replaced by another DNA-binding protein. To address the first possibility, we analyzed the DNA-binding properties of purified *Yl*Tay1p using all variants of telomeric repeats found in the *Yarrowia* clade species as a substrate (Fig. 2). The electrophoretic-mobility shift assay (EMSA) experiments demonstrated that *Yl*Tay1p binds most of these sequences with affinity comparable to the natural telomeric repeats of *Y*. *lipolytica*. Slightly decreased affinity toward the probe was observed only in case of *C*. *hispaniensis* repeat. Altoghether, our data indicate that *Yl*Tay1p binds with comparable affinities to the telomeric sequences containing motif 5′-TT—AGGG-3′.

### Deletion of the putative TER locus affects telomere length in *Y*. *lipolytica*

**Figure 3:**
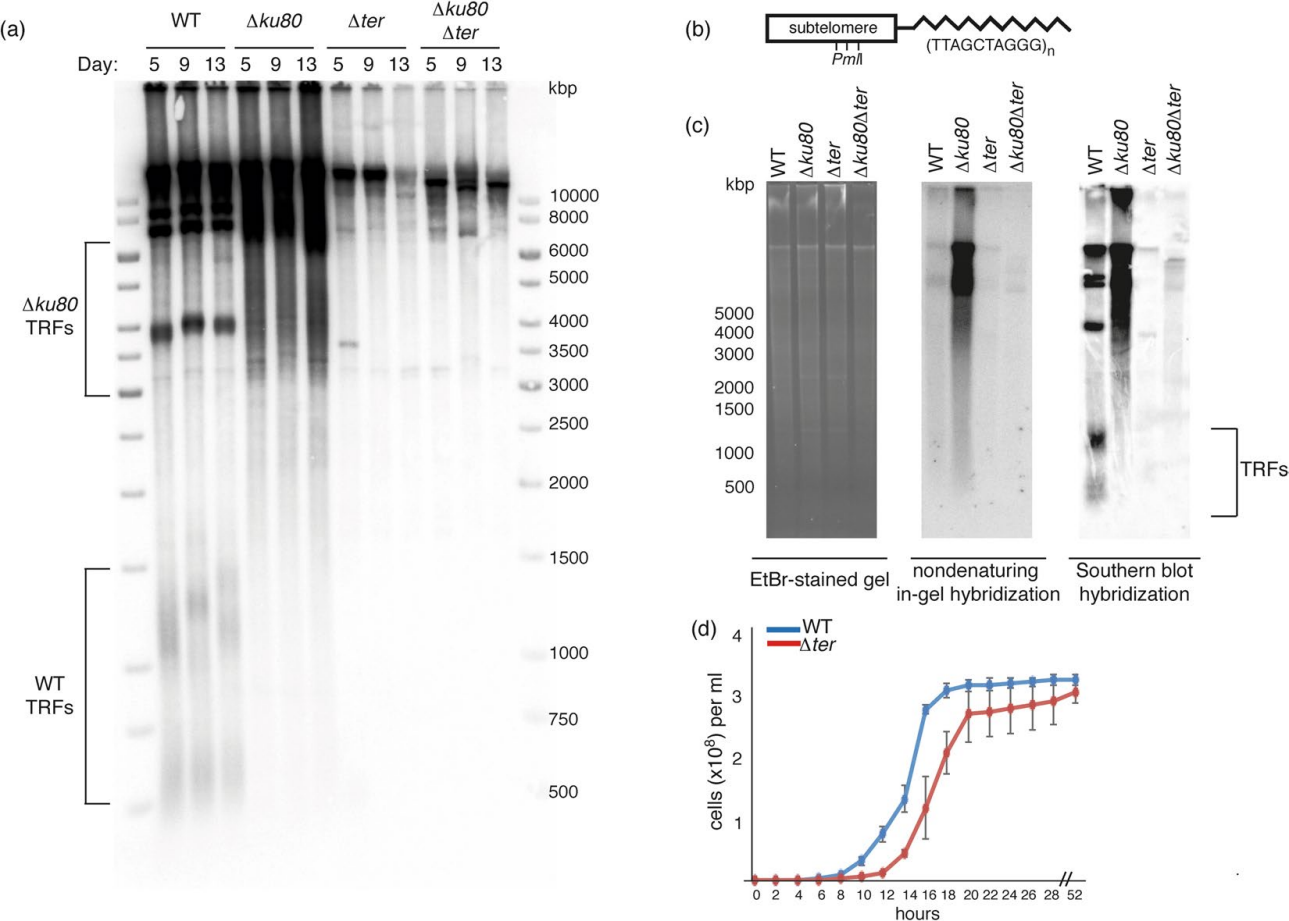
Deletions of TER and/or *YlKU80* genes affect telomere length, single-stranded telomeric overhang and growth in *Y*. *lipolytica*. (**a**) The strains with indicated genotypes were passaged 2 times (each passage took 4 days) and the length of telomeres was assessed by TRF analysis. (**b**) Scheme of a chromosomal end containing a subtelomere with multiple *Pml*I restriction sites (modified from Kinsky et al., 2010). (**c**) The length of telomeric overhangs was measured by in-gel hybridization under nondenaturing conditions using a C-rich telomeric probe (central panel) and compared with the hybridization signal obtained by standard Southern blot hybridization (right panel). Ethidium bromide stained gel served as a loading control (left panel). (**d**) The comparison of growth rates of wild-type (WT) and Δ*ter* strain. The growth curve of Δ*ter* strain starts 5 days (~30 generations) after the transformation of cells with the deletion cassette.

To assess the phenotype of *Y*. *lipolytica* cells lacking functional telomerase RNA, we constructed a strain (∆*ter*) with the entire TER locus replaced by a deletion cassette containing the *URA3* selection marker. In parallel, we deleted the TER locus in a ∆*ku80* strain lacking the functional *YlKU80* gene^61^, whose product was shown to be involved in telomere maintenance in several model organisms^62–65^. The resulting four strains (WT, ∆*ku80*, ∆*ter* and ∆*ku80*∆*ter*) were subjected to the measurement of their telomere length using telomere restriction fragment (TRF) analysis. As reported earlier, the length of TRFs in the WT strain after digestion with *Pml*I varies between 500 and 1500 nt^52^ (Fig. 3a,b). The nature of longer fragments hybridizing to the telomeric probe is unclear. They may represent population of DNA fragments from chromosomal ends lacking *Pml*I site in the vicinity of telomeric tract and/or nontelomeric DNA fragments containing telomere-like repeats that are resistant to BAL-31 treatment^52^.

In contrast to the WT, the TRFs are absent in ∆*ter* strain and a complete loss of telomeric DNA was observed already after the first passage (~25 generations). The telomeres in the ∆*ku80* strain are prolonged and heterogeneous, similar to the situation in mutants lacking active Ku heterodimer in *C*. *albicans* or the plant model *Arabidopsis thaliana*^66,67^. The double mutant ∆*ku80*∆*ter* exhibited loss of telomeres comparable to ∆*ter*, indicating that the telomeres in *∆ku80* strain are maintained by telomerase.

The length of the 3′ telomeric overhang of *Y*. *lipolytica* was measured via in-gel hybridization of non-denaturated TRFs to oligonucleotide probe (Fig. 3c). The weak signal obtained for the WT suggests that similarly to other yeast species (e.g. *S*. *cerevisiae*), the overhang is relatively short. The increased size and heterogeneity of TRFs in ∆*ku80* mutant are accompanied by an increased length of 3′ overhang. In contrast, the strains lacking the TER locus exhibited a complete loss of telomeric single-stranded DNA, regardless of the absence or presence of a functional Ku70/80 complex.

The deletion of TER locus also resulted in slightly delayed (~3 hours compared with the WT) onset of the exponential growth phase in ∆*ter* strain, probably due to critical telomere shortening and senescence of a subpopulation of cells (Fig. 3d). The rapid recovery of the ∆*ter* cells from this crisis suggests that there is an effective back-up system activated in *Y*. *lipolytica* cells lacking functional telomerase. The double mutant ∆*ku80*∆*ter* exhibited a more pronounced growth defect (data not shown), probably caused by the lack of end-protection by Ku70/80.

### Comparative and functional analysis of TER sequences from the *Yarrowia* clade species

**Figure 4:**
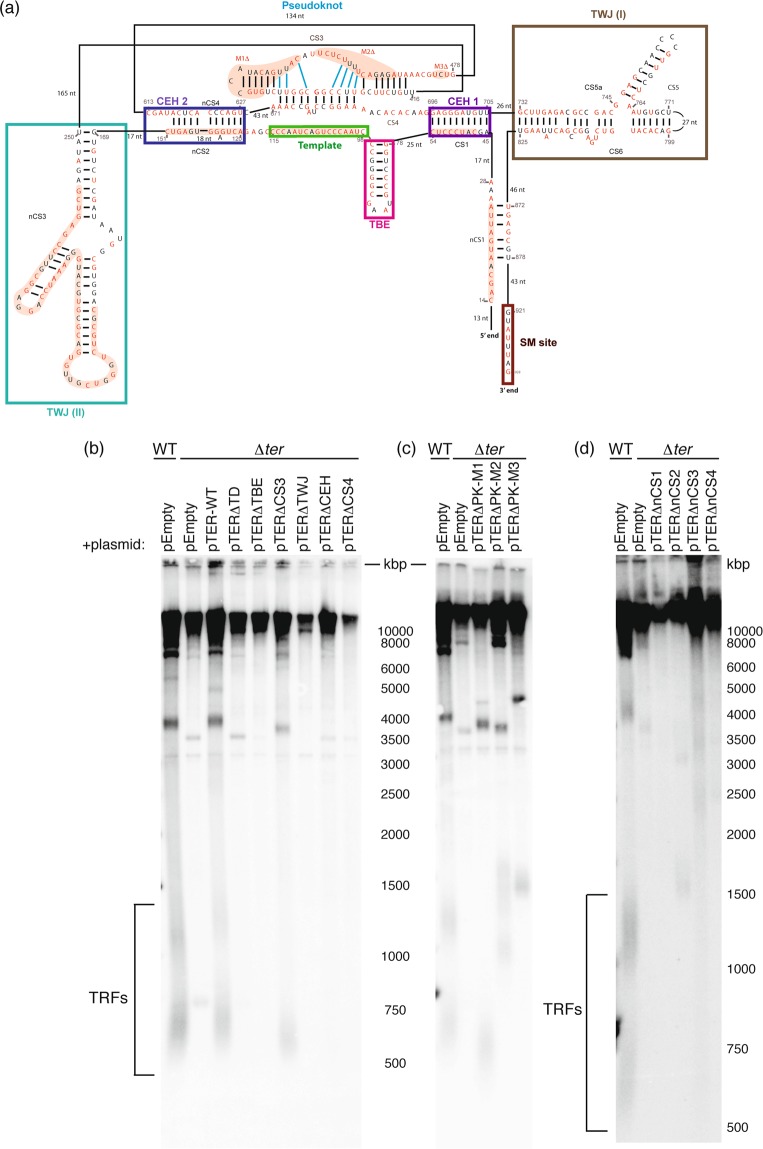
Effect of the deletions of conserved and novel functional elements on the ability of TER to complement Δ*ter* mutation in *Y*. *lipolytica*. (**a**) A simplified scheme of *Y*. *lipolytica* TER with highlighted conserved domains and sequences subjected to functional analysis (for a more detailed structure of TER see Supplementary Fig. S5). (**b**–**d**) Wild-type (WT) and Δ*ter* strains were transformed with the plasmid constructs bearing the TER locus or its deletion variants (deletions of specific sequences are indicated), followed by the TRF analysis to test the ability of the plasmids to restore telomeric fragments in Δ*ter* mutant.

For the identification of putative functional elements involved in telomerase function, we aligned the nucleotide sequences of TER genes from 10 species using ClustalX^68^ (Supplementary Fig. S4; TERs of *Y*. *phangngensis*, *Y*. *alimentaria* and *C*. *hispaniensis* are too divergent, only allowing the alignment of regions corresponding to template domain, pseudoknot and the TWJ). Among these sequences, the level of conservation was high enough for generating a reliable alignment for 1–930 nt of *Y*. *lipolytica* TER, but beyond this point the sequences diverged too much and could not be aligned reliably. Furthermore, we identified an imperfect Sm site at position 920 nt, suggesting that longer transcripts could be processed to a shorter mature RNA with an Sm site close to its 3′ end, which is consistent with the results of Northern blot analysis (data not shown). For the purpose of covariation-based structure prediction we used the alignment that ends at the potential Sm site as an input for the *RNAalifold*^69^. Then we used the resulting *RNAalifold*-predicted helices as constraints for *Mfold*^70^ (Supplementary Fig. S5, Fig. 4a). Using this approach, we identified four conserved elements of the core structure of TER, known in yeasts and vertebrates: template, template-boundary element (TBE), a triple-helix containing pseudoknot (Supplementary Fig. S6), and a core-enclosing helix upstream of the template (CEH1). Interestingly, we identified another conserved helix downstream of the template, termed core-enclosing helix 2 (CEH2). Both CEH1 and CEH2 position the pseudoknot across the template providing further support for the role of the pseudoknot in regulating the template copying by telomerase. In addition to the template domain, we identified the conserved TWJ (Supplementary Fig. S7) and a novel element composed by conserved sequence nCS3, forming another TWJ with two stem-loops (TWJ (II)).

To perform functional analysis of the identified structural elements, we first constructed an episomal plasmid (pTER) bearing the entire intergenic sequence located between *FRE2* and *FRE3* genes of *Y*. *lipolytica*, carrying the TER locus and both 5′ and 3′ flanking sequences ensuring the presence of regions necessary for the regulation of transcription. Transformation of ∆*ter* strain by pTER resulted in the restoration of standard-length TRFs, indicating that the telomere defect due to TER deficiency is reversible and can be complemented by ectopic expression of TER (Fig. 4b). Subsequently, we used this system for testing the ability of deletion variants of TER lacking specific structural elements to restore the TRFs in the ∆*ter* strain. The deletion of template domain, template-boundary element, core-enclosing helix 1, TWJ and CS4 part of the pseudoknot caused a complete loss of the corresponding TER variants ability to restore TRFs. On the other hand, the variant lacking the CS3 sequence partially retained this ability. The restored TRFs in these cells were slightly shortened, but maintained throughout several (~120) cell divisions (Fig. 4b).

To characterize the elements of the pseudoknot structure in more detail, we analyzed three TER variants, lacking specific parts of the CS3 sequence (Fig. 4c). The deletion of motif 1 (M1), which includes a non-conserved stemloop (nucleotides 438–452), led to similar reduction of the TRF size as the deletion of the entire CS3 domain, suggesting this structure is crucial for the proper arrangement of CS3. Interestingly, the deletion of motif 2 (M2, nucleotides 455–468) resulted in prolonged TRFs (1000–1900 bp), implying that this mutation altered the spatial organisation of TER and increased the overall telomerase activity. The deletion variant lacking motif 3 (M3, nucleotides 484–486), which lies 8 nucleotides downstream from the 3′ end of CS3, also resulted in elongated TRFs (~1800 bp). Based on these data, we suggest that the pseudoknot might be involved in both positive and negative regulation of telomerase activity in *Y*. *lipolytica*.

Next, we performed the functional analysis of TER variants lacking the putative novel functional elements (nCS1–4). All four of them are located in the conserved part of TER (Supplementary Fig. S4, Fig. 4a) and their secondary structure is preserved in different species. The first element (nCS1) is located ~15 nt downstream from the 5′ end of the molecule, forms a stem structure and its deletion caused a loss of TRFs comparable to the ∆*ter* strain (Fig. 4d). In the structure prediction (Supplementary Fig. S5), the nCS2 element is paired with nCS4, forming a helix (CEH2) which stabilizes the core structure of TER. Deletion of either nCS2 or nCS4 was predicted to alter this structure and indeed abolished the ability of corresponding TER variant to restore standard TRFs in ∆*ter* strain (Fig. 4d). The deletion variant of TER lacking nCS3 also failed to restore normal TRFs pattern, suggesting that this structure plays an essential role in telomerase activity (Fig. 4d). Thus all four tested elements seem to be essential for proper telomerase function. Interestingly, some mutations in TER caused telomere elongation, indicating that their detailed analysis might be instrumental in shedding more light on telomerase regulation.

### Sequences involved in the regulation of expression and stability of TER in *Y*. *lipolytica*

**Figure 5:**
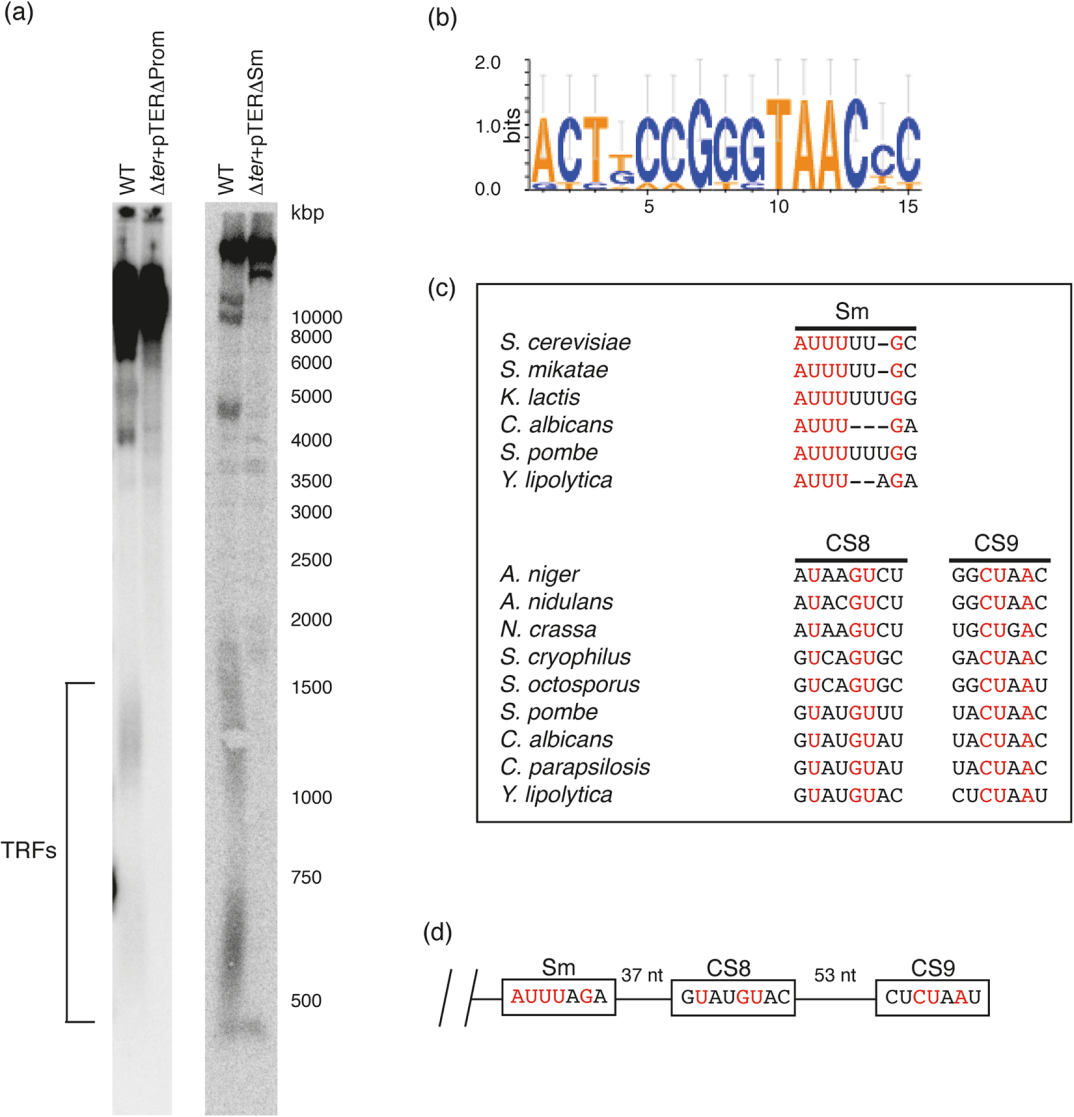
Sequences at the 5′ and 3′ region of TER are involved in the regulation of expression and stability of TER in *Y*. *lipolytica*. (**a**) Wild-type (WT) and Δ*ter* strains were transformed with the plasmid constructs carrying TER lacking putative promoter (Prom) or Sm site (Sm), followed by the TRF analysis to test the ability of the plasmids to restore telomeric fragments in Δ*ter* mutant. (**b**) Sequence logo representing consensus sequence of TER promoter in *Yarrowia* clade species. (**c**) Comparison of the sequences of Sm site, CS8 and CS9 from *Y*. *lipolytica* TER with those of other yeast models. (**d**) Scheme representing the 3′ end of *Y*. *lipolytica* TER with indicated positions of putative conserved elements.

The comparative analysis of TERs from the *Yarrowia* clade species also revealed a conserved sequence lying ~40 nt upstream of the transcription start site, representing a putative TER promoter (Supplementary Fig. S4). This sequence includes the conserved motif 5′-TAAC-3′, which is present in all of the 10 aligned TERs (Fig. 5b). In agreement with the prediction that this sequence is functionally important, the plasmid construct bearing the complete TER sequence, yet lacking the putative promoter was unable to restore the TRFs in ∆*ter* strain (Fig. 5a).

An Sm consensus sequence, which is bound by the Sm proteins and facilitates the assembly of the telomerase ribonucleoprotein complex, was found at the 3′ ends of TERs of filamentous fungi, budding and fission yeast^18,38,41,45,46^. Furthermore, consensus 5′ splicing site and branch point were found in *Candida*, *Schizosaccharomyces* and *Aspergillus* species, and shown in *S*. *pombe* to facilitate 3′ end-processing by a partial splicing reaction^18,45,46^. At the predicted 3′ end of *Y*. *lipolytica* TER, a consensus Sm site was found with sequence similar to the Sm site of *C*. *albicans* TER (Fig. 5c, Supplementary Fig. S5). The position of this sequence, consistent with the RNA-seq results (Supplementary Fig. S1), shows that detectable TER transcripts are about 950 nt long. The *Yarrowia* species TER sequences downstream of CS6 are too diverged for a reliable multiple sequences alignment, thus we could not obtain phylogenetic support for this prediction. However, the deletion variant of TER lacking this sequence was unable to complement the loss of TRFs in ∆*ter* strain (Fig. 5a), suggesting that it is a functional Sm site required for the proper processing of TER and the assembly of active telomerase *in vivo*. Importantly, homologs of all 7 human Sm proteins known to bind the Sm site and stabilize the TER molecule (SNRPB – YALI0D14102p, SNRPD1 – YALI0A04961p, SNRPD2 – YALI0F06644p, SNRPD3 – YALI0A19030p, SNRPE – YALI0D01155p, SNRPF – YALI0A05423p, SNRPG – YALI0F30426p) were also identified in the genome, suggesting the mechanism of TER stabilization is conserved in yeasts.

We also identified several candidate sequences for putative 5′ splice sites (CS8) and for branch point (CS9) in the *Y*. *lipolytica* TER. The best CS8 candidate downstream of the Sm site is nearly identical to CS8 from *S*. *pombe* and is followed by a degenerate CS9 motif (Fig. 5c,d). Candidate CS8 and CS9 sequences were also found in the other *Yarrowia* species TERs, but at different distances from CS6, suggesting that the 3‘ end of the mature TER transcripts vary significantly.

### Transcriptome response to the loss of telomerase activity

Diverse telomeric repeats recruit not only diverse DNA-binding proteins, but indirectly influence also other proteins associated with telomere, including DNA repair factors, replication-assisting proteins and factors responding to cellular stress. The vertebrate-like telomeric repeat of *Y. lipolytica*, bound by different proteins, could thus trigger different stress response than other telomeric sequences found in budding and fission yeasts. To adress this possibility, we studied the transcriptomic response to telomere loss of ∆*ter* strain in this yeast. This analysis led to the identification of 111 differentially expressed genes (DEGs) annotated in the H222-S4 genome. Among these DEGs, only one has a homolog among the known telomere-related proteins. YALI0C17061g, up-regulated in the ∆*ter* strain, is a homolog of PARP1 and PARP3 proteins that are involved in poly(ADP-ribosylation) of TRF2, telomere length regulation and telomere integrity maintenance in human cells^71–73^. In addition, PARylation executed by PARPs mediates DNA damage response at DNA breaks and participates in oxidative stress response (OSR)^74^. Interestingly, other genes putatively involved in the OSR were also detected as over-expressed in ∆*ter* strain (Table 1). These involve YALI0C16621g, a predicted superoxide dismutase (SOD) gene, YALI0E02266g, containing a Cu-Zn binding SOD domain and YALI0B13200g, containing a leucine zipper domain of YAP family transcription factors that are involved in stress responses. Moreover, the overexpression in ∆*ter* was also detected for YALI0C17567g, a homolog of *DDR48*, a DNA-damage response factor linked to the OSR and DNA replication stress in *S. cerevisiae*^75^. An additional functional enrichment analysis has shown that the genes differentially expressed in ∆*ter* strain can be grouped in four major functional categories (Table 2). Previously, a number of environmental stress response genes have been found to be overexpressed in both *S. cerevisiae* and *H. polymorpha* TER deletion mutants. However, OSR has been observed in telomerase mutant of *H. polymorpha* (with telomeric repeat 5′-GGGTGGCG-3′ and undescribed telomere-binding proteins)^76^, mouse cells lacking telomerase^77^ and human cells lacking specific components of telomerase holoenzyme^78^, but not in *S. cerevisiae*^79^. In conclusion, these results suggest that telomerase loss in *Y. lipolytica* induces OSR, a feature it shares with some yeasts and higher eukaryotes.

**Table 1:** Several genes overexpressed in ∆ter strain are possibly involved in the oxidative stress response.

DEG	Putative function/functional domain	Fold change (∆*ter*/WT)
YALI0C17061g	Poly(ADP-ribose) polymerase	2.07
YALI0C16621g	mitochondrial superoxide dismutase	7.62
YALI0E02266g	Cu-Zn binding SOD domain	2.19
YALI0B13200g	YAP transcription factor leucine zipper domain	2.98
YALI0C17567g	DNA damage-responsive protein 48	2.82

**Table 2:** A functional enrichment analysis has shown several pathways possibly induced in ∆ter strain.

Functional enrichment category	Number of genes
Transporters/transmembrane proteins	36
Iron metabolism & dehydrogenases	13
Aldo/keto reductases and oxidases	9
Secondary metabolites production	7

The categories are listed according to the number of included DEGs. Category “Transporters/transmembrane proteins” was reported as two separate enrichment groups by DAVID analysis. As the DAVID software does not provide the enrichment groups with labels, the names of the categories were chosen arbitrarily, to fit the contained functional terms.

## Discussion

The species of the *Yarrowia* clade provide an excellent opportunity to assess co-evolution of the components of telomere maintenance system. Remarkably, while they are relatively closely related, they do exhibit a clear diversification of their telomeric repeats. Taking advantage of these characteristics we addressed two questions: (1) how are the changes in the primary sequence of telomeres reflected by the DNA-binding properties of telomere-binding proteins, and (2) what are the common structural features of the RNA component of telomerase that are required for its activity *in vivo*. Answering these questions is relevant not only for a distinct group of fungi, but is also important for gaining general insights into the evolution of eukaryotic telomeres.

The double-stranded region of *Y*. *lipolytica* telomeres is bound by Tay1 protein, which is in many ways different from the other yeast dsDNA-binding telomeric proteins. It possesses 2 Myb domains and binds the canonical mammalian 5′-TTAGGG-3′ repeats with higher affinity than the telomeric sequence of *Y*. *lipolytica*^54^. It is therefore possible that the insertion in the template domain of TER that changed the sequence of telomeric repeat in the common ancestor of the *Yarrowia* clade (and possibly all Saccharomycotina) was tolerated while Tay1p was still able to bind the telomeric repeat, providing it retained the core of the ancestral motif as in case of *Y*. *lipolytica* (5′-TTAGTCAGGG-3′). According to this scenario, the inner four nucleotides (5′-AGTC-3′) represent a part of the telomeric repeat whose alterations should not have a substantial effect on Tay1p binding. Importantly, all 13 species contain an ortholog of *Yl*Tay1p whose Myb domains underwent only minor (if any) amino acid substitutions (Supplementary Fig. S3), suggesting the crucial role of this protein in telomere maintenance might be conserved in the whole clade. Particularly interesting is *C*. *hispaniensis*, whose telomeric repeat encountered substitutions in all four positions of the spacer region plus G-to-A substitution in the 9th position of the repeat affecting the core 5′-TT—AGGG-3′ sequence (Fig. 1). *Yl*Tay1p exhibited a decreased affinity toward *C*. *hispaniensis* telomeric repeat (Fig. 2), supporting the importance of the intact core sequence for an optimal Tay1 binding. However, similarly to other species of the *Yarrowia* clade, *C*. *hispaniensis* seems to lack the homologs of *Sp*Taz1 or *Sc*Rap1 proteins that exhibit flexible binding to telomeric repeat variants. It is thus likely that even though the affinity for an altered telomeric repeat is decreased, it is still sufficient for the Tay1 protein to fulfill the crucial telomeric functions. According to our hypothesis, more extensively altered telomeric repeats of other yeast species might not have been recognized by Tay1p homologs and as a result, evolutionary novel telomeric proteins such as Rap1 or Taz1 emerged^21^, while Tay1p homologs (such as *Sp*Teb1) were either lost or retained to act as transcription factors recognizing TTAGGG-like repeats in regulatory sequences of their target genes^23^.

The rapid loss of TRFs in the *Y*. *lipolytica* strain lacking putative TER locus confirmed that this sequence is indeed transcribed into functional telomerase RNA (Fig. 3a). The complete loss of TRFs was observed in very early generations (less than 50 cell divisions), unlike what has been observed in *S*. *cerevisiae* ∆*tlc1* or *S*. *pombe* ∆*ter1* mutants, where even after more than 100 generations, short TRFs can be detected^24,26^. This phenotype is reminiscent of the situation in *Y*. *lipolytica* ∆*est2* cells lacking the catalytic subunit of telomerase^52^ and suggests that telomerase dysfunction results in very rapid telomere shortening, accompanied by the activation of a back-up system which allows a subpopulation of cells to overcome the early growth crisis and escape senescence. Since both ∆*ter* and ∆*ku80*∆*ter* strains are able to overcome the crisis, the back-up system does not require the activity of Ku70/80 heterodimer. The complete loss of detectable TRFs in both strains also suggests that this process does not include telomerase-independent amplification of telomeric DNA. In contrast to the ∆*ku80*∆*ter* strain of *Y*. *lipolytica*, *S*. *cerevisiae* cells lacking both *TLC1* and *YKU80* genes are not viable, suggesting the mechanism of chromosome end maintenance in ∆*ter* mutant of *Y*. *lipolytica* is different from that of ∆*tlc1* mutants of *S*. *cerevisiae*^62^. On the other hand, ∆*ku80* mutants of *K*. *lactis* expressing a modified allele of TER (ter1-4LBsr) and thus introducing altered repeats into its telomeric tract are viable, which is in line with our observations^65^. In agreement with previous studies on ∆*est2* strain^52^, it is possible that in a *Y*. *lipolytica* ∆*ter* strain, chromosomal ends are maintained via amplification of subtelomeric repeats by homologous recombination. In ∆*ku80* strain, TRFs appear to be prolonged and heterogenous (Fig. 3a), similarly to *C*. *albicans* ∆*ku70/*∆*ku70* strain^66^, suggesting that Ku70/80 heterodimer is a negative regulator of telomere length in *Y*. *lipolytica*. In line with this hypothesis is the observation that ∆*ku80*∆*ter* strain exhibits the same telomeric phenotype as the single ∆*ter* strain (Fig. 3a).

The telomeric 3′ overhang is prolonged in the ∆*ku80* strain of *Y*. *lipolytica* (Fig. 3c), which is most probably due to increased nucleolytic degradation of telomeric C-strand^62,63^. This phenotype is also reminiscent of *S*. *cerevisiae* strains lacking functional Ku70/80 heterodimer, whose ssDNA overhangs are prolonged throughout the cell cycle and occupied by an increased amount of Cdc13p molecules^80^. In ∆*ter* and ∆*ku80*∆*ter* strains, the loss of TRFs is accompanied by a complete loss of telomeric ssDNA, suggesting the alternative mechanism of chromosome-end maintenance does not involve preserving the 3′ overhang composed of telomeric repeats.

To further investigate how the divergence of telomeric repeats of the *Yarrowia* clade species co-evolved with the corresponding RNA components of telomerase, we performed a bioinformatic and functional analysis of TERs from all 13 species. The results underlined the overall diversity of these structures observed in different sets of organisms^18,41,81^ (Supplementary Fig. S8). Even the 10 species, whose TERs are similar enough for sequence alignment, exhibited major differences in its length and sequence. As expected, the most conserved of the entire TER is the template domain, where we were able to identify the conserved motif 5′-TAACCC-3′ (which presumably serves as the template for synthesis of one vertebrate-type telomeric repeat) in each of the 10 aligned TERs. Strong conservation can also be observed in the regions representing the TWJ and the CS4 subunit of pseudoknot. Interestingly, the sequence of CS3 is only partially conserved with several variants of the central part and it is not essential for the telomerase function. The TRFs observed after transformation of ∆*ter* strain with plasmid bearing TER variant lacking CS3 are shorter than the wild-type TRFs, but they are maintained throughout several cell divisions, suggesting this deletion variant of TER is able to partially associate with catalytic subunit of telomerase (Fig. 4b).

There are also several features typical for the conventional yeast TERs missing in the TER of *Y*. *lipolytica* (and also TERs from other species of the *Yarrowia* clade). The absence of Est1-binding arm is in agreement with the fact that there is no clear homolog of the *ScEST1* gene in the genome of *Y*. *lipolytica* and other *Yarrowia* species. These data imply that the connection between telomeric DNA and telomerase is mediated by a yet unknown and possibly unique set of regulatory proteins in these species. The 5′ arm with the binding site for Ku heterodimer, typical for *Saccharomyces* species and *Candida glabrata*^82^ is also absent in *Yarrowia* clade TERs, although Ku70/80 is a key regulator of telomere maintenance, suggesting the association of Ku70/80 with telomerase is indirect and might involve other interacting partners.

Another feature, essential for telomerase activity in *S*. *cerevisiae* is the P3-like domain. In *TLC1* RNA, this domain provides the binding site for Pop6 and Pop7 proteins, facilitating the assembly of the telomerase Est1-Cdc13 recruitment module^43,83^. In *Y*. *lipolytica* TER, we were not able to identify a homologous domain by a simple sequence alignment, however, the nCS1 element, which is essential for telomerase activity, shares several similarities with this structure. Specifically, the secondary structure prediction shows that similarly to *S*. *cerevisiae* P3-like domain, nCS1 also forms a bulged stem structure with conserved A-U and G-C pairs at the base of the stem (Supplementary Fig. S5)^43^. This indicates that nCS1 element might be involved in the assembly of *Y*. *lipolytica* telomerase in a similar way as the P3-like domain of *TLC1* RNA. Another candidate for the P3-like domain in *Y*. *lipolytica* TER is the TWJ (II), which lies in a similar position as the P3-like domain of *S*. *cerevisiae TLC1* RNA. However, its role in telomerase assembly is yet to be tested experimentally. On the other hand, the protein composition of telomerase recruitment module in *Y*. *lipolytica* is probably different from that of *S*. *cerevisiae*, since there is no clear ortholog of *ScPOP6* or *ScPOP7* gene present in its genome (YALI0E29007g is the ortholog of *ScPOP1*).

We also assessed the transcriptome response to the lack of TER using the RNA-seq analysis. Surprisingly, the analysis did not reveal a change in expression of a distinct group of telomere-related genes such as *TAY1*, possibly reflecting the essential, non-telomeric roles of Tay1p^53^ (Table 2). However, several genes possibly involved in OSR were found to be up-regulated in the ∆*ter* strain (Table 1). This may point to a more conserved role of telomere damage triggering the OSR—in human cells mediated by p53 and p21 pathway^84^. Although the homologs of p53 and p21 are not present in the *Y*. *lipolytica* genome and many other genes involved in the OSR are not affected, it would be interesting to further elucidate the variability of factors mediating the OSR in species with different telomere composition.

## Conclusion

Our study, aimed at comparative analysis of the *Yarrowia* clade species, in essence caught evolution of telomeres in action. We have shown that changes in telomeric repeats are constrained by the DNA-binding properties of the Tay1 protein, which tolerates substitutions within the region outside the core 5′-TTAGGG-3′ motif. It is possible that *C*. *hispaniensis*, a species with a more diverged sequence of the telomeric repeat is just at the tipping point of the phylogenetic tree where Tay1-like telomeric proteins were replaced by more flexible telomere binding factors allowing further diversification of the telomeric sequences. Additionally, our work demonstrated that comparative studies of TERs such as the one reported here are still worthwhile as they can lead to identification of novel conserved structural motifs whose functions are important for telomerase activity *in vivo*.

## Materials and Methods

### Yeast strains

In this study, *Y*. *lipolytica* strain H222-S4 (*MAT*A, *ura3-302 SUC2*) (Barth and Gaillardin, 1996) was used as the wild type. Wild type strain and H222-SW6 strain (*MAT*A, *ura3-302 SUC2* ∆*ku80*) lacking *YlKU80* gene (*∆ku80*)^61^ were kindly provided by Gerold Barth (Technische Universität Dresden, Dresden, Germany). Both strains lacking the TER locus (∆*ter*, ∆*ku80*∆*ter*) were derived from H222-S4 and H222-SW6 strains, respectively, using the strategy described below.

### Cultivation and media

For each experiment, yeast cells were cultivated in YPD [1% (w/v) yeast extract, 2% (w/v) Bacto Peptone, 2% (w/v) glucose] or SD [0.17% (w/v) yeast nitrogen base, 0.5% (w/v) ammonium sulfate, 2% (w/v) glucose] medium at 28 °C with constant aeration. Cultures used for construction of growth curves started at 10^5^ cells/ml in YPD medium, 5 µl aliquotes were collected every 2 hours and cells were counted in a Bürker chamber. Each experiment was carried out in triplicate, the total amounts of cells were counted separately for the three independent cultures and the indicated numbers were calculated as means. The error bars represent the standard deviation.

### Tay1 protein purification and electrophoretic mobility shift assays (EMSA)

Recombinant Tay1 protein was purified as described previously^53^. The 10 µl EMSA reactions contained 20 mM HEPES-NaOH (pH 7.3), 100 mM NaCl, purified Tay1p at indicated concentrations and 16 nM dsDNA probe, prepared as follows: the G-rich single-stranded oligonucleotides (sequences are listed in Supplementary Table S1) were labelled at 5′ end by T4 polynucleotide kinase (Thermo Scientific) using [γ-^32^P]ATP (Hartmann Analytic), mixed with 3-fold molar excess of the unlabelled complementary oligonucleotide, heated for 10 min at 100 °C and slowly cooled down at room temperature to allow efficient formation of the double-stranded probes. The reaction mixtures were incubated for 10 min at room temperature, afterwards the glycerol was added to the final concentration of 4.5% (v/v) and the samples were loaded on 6% polyacrylamide gel in 0.5x TBE buffer [40 mM Tris-HCl (pH 8.3), 45 mM boric acid, 1 mM EDTA]. The electrophoresis was performed in cold 0.5x TBE buffer at 20 mA per gel for 20 min. The gels were fixed with 10% (v/v) methanol, 10% (v/v) acetic acid for 10 min, dried and exposed to a phosphor screen. Signal was detected using Personal Molecular Imager FX (BioRad). The contrast of images was adjusted using Adobe Photoshop 12.0.

### Construction of the ∆*ter* and ∆*ku80*∆*ter* strains

The entire TER locus including ~1200 bp long upstream and downstream flanking regions was amplified (for the list of oligonucleotides see Supplementary Table S1) and cloned into pDrive cloning vector (QIAGEN). The resulting plasmid was digested with restriction enzymes *Nde*I and *Bgl*II to remove the transcribed part of the locus and gel-purified. The *URA3* gene was PCR-amplified using primers containing the restriction sites for *Nde*I and *Bgl*II and ligated with the linearized plasmid. The full-length disruption cassette was PCR-amplified and used for transformation of H222-S4 and H222-SW6 cells. Transformants were selected after 3 days (~20 generations) of incubation at 28 °C on SD media lacking uracil and the disruption of the TER locus was verified by PCR and sequencing.

### TRF analysis

Total genomic DNA (gDNA) was isolated as described by Barth and Gaillardin^85^. 3 µg of gDNA were digested using 6 U of *Pml*I overnight. The fragments were separated in 1% (w/v) agarose gel for 16 hours at 1.6 V/cm and stained with 0.5 µg/ml ethidium bromide solution for 20 minutes (stained gel served as a loading control). The gel was then incubated for 40 minutes in denaturation solution (1.5 M NaCl, 0.5 M NaOH), 30 minutes in neutralization solution (1.5 M NaCl, 0.5 M Tris, pH 7.4) and 30 minutes in 20x SSC (3 M NaCl, 0.3 M Na-citrate, pH 7.0). The DNA was then transferred to Immobilon NY+ membrane (EMD Millipore) with a VacuGene XL blotter (GE Healthcare) in 20x SSC and fixed by incubating the membrane at 80 °C for 1 hour. The membrane was pre-hybridized for 30 minutes at 65 °C in Church buffer [0.25 M sodium phosphate buffer (pH 7.2), 1 mM EDTA] and hybridized at 65 °C overnight in the same buffer containing 50 ng of denaturated telomere-specific probe (YlTEL probe 1; Supplementary Table S1) labelled with [α-^32^P] dCTP (Prime-a-Gene® Labelling System, Promega). The membrane was washed once with wash buffer I [0.15 M NaCl, 15 mM Na-citrate, 0.1% (w/v) SDS] for 20 minutes at room temperature and once with wash buffer II [7.5 mM NaCl, 0.75 mM Na-citrate, 0.1% (w/v) SDS] for 1 hour at 50 °C. The signal was detected by Personal Molecular Imager FX (BioRad). The contrast of images was adjusted using Adobe Photoshop 12.0.

### In-gel hybridization

1.5 μg of gDNA digested with *Pml*I was separated in 0.7% (w/v) agarose gel for 16 hours at 60 V. The gel was placed on a double-layer of Whatman paper and mounted on a Gel Dryer (model 583, Bio-Rad). Drying was carried out for 15–20 minutes at room temperature. The dried gel was sealed in a plastic bag and hybridized with 50 ng of oligonucleotide probe (YlTEL probe 2; Supplementary Table S1) end-labelled with [γ-^32^P] ATP in the hybridization buffer (5x SSC, 2.5x Denhardt’s solution, 50 mM pyrophosphate, 1 mM Na_2_PO_4_, 0.4 mM ATP, 20 µg/ml salmon sperm DNA) overnight at 37 °C. After the removal of excess hybridization buffer, the gel was washed 3–4 times for 30 minutes in 0.25x SSC at room temperature with agitation, sealed in a bag and exposed to a phosphor screen. Afterwards, the gel was subjected to a denaturing Southern blot in order to visualize the total telomeric DNA. First, the gel was incubated for 10 minutes in 0.25 M HCl, then for 45 minutes in denaturation solution and 5 minutes in 0.4 M NaOH. The DNA was transferred to a Hybond-XL nylon membrane (GE Healthcare). After the transfer, the membrane was pre-hybridized for 1 hour in Church buffer and hybridized overnight at 50 °C with 50 ng of YlTEL probe 2. The membrane was washed for 20 minutes at room temperature in 2x SSC and exposed to a phosphor screen. The contrast of images was adjusted using Adobe Photoshop 12.0.

### RNA-seq analysis of ∆*ter* mutant

Three independent cultures of both wild type and ∆*ter* yeast cells were cultivated in 5 ml liquid YPD medium until the exponential phase (OD_600_ = 0.6–0.8). Afterwards, total RNA was isolated using Direct-zol^TM^ RNA miniprep kit (Zymo Research) and the quality of the RNA was determined by Agilent 2100 Bioanalyzer (Agilent) using RNA 6000 Nano Assay (Agilent). The RNA integrity number (RIN) of all samples was assessed to be 9.6–9.8. For every sample, the library of oriented reads with single reads of 75 bp (TruSeq Stranded mRNA, Illumina) was prepared from 1 µg of isolated RNA and the libraries were analyzed by NextSeq550 apparatus (Illumina). The six obtained libraries were named WT1 to WT3 and TER1 to TER3. The sequenced libraries were first cleaned with Trimmomatic tool (v. 0.38)^86^ to clip sequencing adapter fragments from reads and to remove low quality regions (Trimmomatic parameter values: ILLUMINACLIP: TruSeq3-SE.fa:2:15:5 LEADING: 5 TRAILING: 5 SLIDINGWINDOW: 5:20 MINLEN: 50). Then, the filtered reads were mapped against H222-S4 reference genome^87^ using HiSat2 aligner tool (v. 2.1.0)^88^ with the following options:–max-intronlen 4000. To report the number of reads associated with each coding gene we used featureCounts tools (v. 1.6.2)^89^ with the option largestOverlap. The reads for the RNA-seq have been deposited at the EMBL-ENA and are publicly available under the accession number PRJEB29941 (https://www.ebi.ac.uk/ena/data/view/PRJEB29941). Differential gene expression analysis was performed under the R environment (v. 3.3.3) using the BioConductor package DESeq2 (v. 1.20.0)^90,91^. A preliminary investigation of the read count matrix revealed important biases in two libraries (TER1 and WT1) leading to inconsistent variance estimations for a large number of genes. Thus, for this analysis, we discarded libraries TER1 and WT1. We considered two criteria to define differentially expressed genes (DEGs): (1) The DESeq2 adjusted p-value was < = 0.001; and (2) Expression variation was at least two-fold between the wild-type and deletion strain. As noted earlier^76^, some of the DEGs may reflect the activation of *URA3*-related metabolic pathways in the *ter1*::*URA3* mutants. Thus, we performed a comparison of the 126 identified DEGs to another DEG dataset from a strain generated by *URA3* integration (*Y*. *lipolytica mhb1*::*URA3*^92^). This analysis yielded 15 shared DEGs, which were excluded from the set and only the remaining 111 DEGs were further analyzed (Supplementary Table S2). To detect telomere-related genes, the DEGs were searched against the SwissProt database (Release 2018_06) with blastp algorithm from BLAST+ 2.2.31^93,94^, E-value threshold <0.0001 and telomere-related proteins were extracted from the list of significant hits based on a set of manually selected GO terms related to telomere biology (Supplementary Table S3). Furthermore, the QuickGO tool (https://www.ebi.ac.uk/QuickGO/) was used to identify DEGs associated with the GO term GO:0006979: response to oxidative stress. An additional DEG functional clustering analysis was performed with the tool DAVID, v. 6.8 (https://david.ncifcrf.gov/^95,96^). A function “Functional annotation clustering” was used to identify the functional enrichment clusters with the defined defaults as a source of functional annotation and the list of DEGs submitted as a gene list. *Y*. *lipolytica* CLIB122 (E150) genome was selected automatically as a background.

### Genome sequencing

For comparative genome analysis, 13 strains of the *Yarrowia* clade were used. Their genomes were sequenced, assembled and annotated at the Micalis Institute, and deposited at the EMBL-ENA (Supplementary Table S4). Different strategies of sequencing and assembly were used. *Y*. *alimentaria* CBS 10151, *Y*. *galli* CBS 9722, *Y*. *phangngensis* CBS 10407, *Y*. *yakushimensis* CBS 10253, and *C*. *hispaniensis* CBS 9996 were sequenced using Roche 454 technology. Single reads were retrieved from a shotgun library and paired-end reads from a 8-kb library for a total coverage ranging from 16.7X (*Y*. *alimentaria*) to 39.46X (*C*. *hispaniensis*). Celera assembler version 6.1 (version 5.3 for *Y*. *galli*) was used. Additional Illumina HiSeq data were used to correct 454 sequencing errors occurring especially at homopolymers for strains CBS 10151, CBS 10253, and CBS 9996. *Y*. *bubula* CBS 12934, *Y*. *deformans* CBS 2071, *Y*. *divulgata* CBS 11013, *Y*. *hollandica* CBS 4855, *Y*. *keelungensis* CBS 11062, *Y*. *oslonensis* CBS 10146, and *Y*. *porcina* CBS 12935 were sequenced using Illumina Solexa technology with a HiSeq2000 system. Both shotgun and 8-kb paired-end libraries were sequenced in paired-end (2 × 100 bp). Raw reads were trimmed with Trimmomatic v.0.32^86^ and Cutadapt v.1.8.3^97^. Assemblies were generated using SOAPdenovo2 v.2.04^98^ with optimal kmer estimated with kmergenie v.1.67^99^. Gap closing was performed using GapCloser v1.12^98^.

### Phylogeny

A set of 97 protein sequences was chosen among the 912 proteins used for a previously published phylogenetic tree drawn for 6 species^60^. The following criteria were used: genes do not possess any introns and have a known function in *Y*. *lipolytica*, genes are singleton in all species of the *Yarrowia* clade, alignment of the protein sequences subsequently cleaned with Gblocks^100^ represents at least 70% of the initial alignment, the resulting alignment is longer than 100 amino acids. The 97 alignments were then concatenated, leading to a 41496-residue alignment. Phylogenetic trees were constructed by maximum likelihood, with PhyML^101^ and a JTT substitution model corrected for heterogeneity between sites by a Γ-law distribution, with four different categories of evolution rates. The proportion of invariable sites and the α-parameter of the Γ-law distribution were optimized according to the data. A bootstrap value was calculated from 100 replicates.

## Supplementary information

Supplementary Information

Dataset 1

## Data Availability

All data generated or analysed during this study are included in this published article (and its Supplementary Information Files).
