# Supplementary material for: Identification of telomerase RNAs in species of the Yarrowia clade provides insights into the co-evolution of telomerase, telomeric repeats and telomere-binding proteins
Source: Sci Rep. 2019 Sep 16;9:13365. doi: 10.1038/s41598-019-49628-6 (PMC6746865; doi:10.1038/s41598-019-49628-6)
Supplement: Supplementary file 1 — Supplementary Information [file 41598_2019_49628_MOESM1_ESM.pdf]

Identification of telomerase RNAs in species of the *Yarrowia* clade provides insights into the co-evolution of telomerase, telomeric repeats and telomere-binding proteins

Filip Červenák[1], Katarína Juríková[1], Hugo Devillers[2], Binyamin Kaffe[3], Areej Khatib[3], Erin Bonnell[4], Martina Sopkovičová[1], Raymund J. Wellinger[4], Jozef Nosek[1], Yehuda Tzfati[3,*], Cécile Neuvéglise[2,*], Ľubomír Tomáška[1,*]

[1]Departments of Genetics and Biochemistry, Comenius University in Bratislava, Faculty of Natural Sciences, Ilkovičova 6, Mlynská dolina B1 and CH-1, 84215 Bratislava, Slovakia; [2] Micalis Institute, INRA, AgroParisTech, Université Paris-Saclay, 78350 Jouy-en-Josas, France; [3]Department of Genetics, The Silberman Institute of Life Sciences, The Hebrew University of Jerusalem, Safra Campus, Jerusalem, 91904, Israel; [4]Department of Microbiology and Infectiology, RNA Group, Faculty of Medicine and Health Sciences, Université de Sherbrooke, Sherbrooke, Québec J1E 4K8, Canada

[*]to whom correspondence should be addressed
lubomir.tomaska@uniba.sk
tzfati@mail.huji.ac.il
cecile.neuveglise@inra.fr

List of Supplementary Information

Supplementary Figure S1. Position of reads from RNA-seq analysis of the TER locus of *Y. lipolytica*.

Supplementary Figure S2. Translocation events resulting in relocalization of TER locus in *Y. hollandica* (S2a) and *Y. phangngensis* (S2b).

Supplementary Figure S3. Comparison of Myb domains of Tay1p homologs in the species belonging to the *Yarrowia* clade. Red letters indicate positions within telomeric repeats and Myb domains, respectively, that underwent substitutions compared with the corresponding sequences in *Y. lipolytica*. Shaded parts of the telomeric repeats indicate conserved positions. Both Myb domains are aligned to Myb domains of human TRF1 and TRF2 proteins. The regions corresponding to helices 1–3 are derived from Court et al. (2005). Asterisks indicate conserved positions.

Supplementary Figure S4. Clustal X alignment of TER orthologs from the *Yarrowia* clade species.

Supplementary Figure S5. Model of a 2D structure of *Y. lipolytica* TER. The positions of conserved sequences (CS) are indicated by brown areas and green text. Blue text is used to describe specific secondary structures. The light-green bases are conserved in at least 9/10 species whose TERs were aligned.

Supplementary Figure S6. Predicted structures of pseudoknots in TER from indicated *Yarrowia* spp. The two conserved sequences forming the pseudoknot (CS3, CS4) are indicated. The red bases are conserved in at least 9/10 species whose TERs were aligned.

Supplementary Figure S7. Predicted structures of three-way junctions in TER from indicated *Yarrowia* spp. The three subunits of the three-way junction (CS5, CS5a, CS6) are indicated. The red bases are conserved in at least 9/10 species whose TERs were aligned.

Supplementary Figure S8. (a) Main features and motifs in TER sequences of Saccharomycotina yeasts. The phylogenetic tree is based on the concatenation of 104 groups of orthologous proteins (40,077 residues) chosen with the criteria defined in Materials and methods. The tree was constructed with PhyML with a LG substitution model corrected by a Γ-law distribution, with four different categories of evolution rates. *Schizosaccharomyces pombe* was used as an outgroup. Branch support was estimated with aLRT non-parametric branch support based on a SH-like procedure, with seaview. Data for Saccharomycetaceae species are from Waldl et al. (2018), Kachouri-Lafond et al. (2009), and the Telomerase Database http://telomerase.asu.edu/; those for the *Candida albicans* clade from Gunisova et al. (2009). KU: ku binding hairpin; T: template region; EST1: Est1 binding site; TWJ: three-way junction; S: Sm binding site; CS1, nCS1, CS3, nCS4 and CS4 refer to

conserved sequences; pseudo: pseudoknot including CS3 and CS4; ? putative pseudoknot with unresolved secondary structure.

Supplementary Figure S8. (b) Schematic models for fungal and vertebrate TERs. In red, elements conserved across all fungal and vertebrate TERs examined. In blue, elements conserved among all fungal or all vertebrate TERs examined. In purple, elements conserved among some but not all groups of fungi. Based on Brown et al. (2007).

Supplementary Table S1. List of oligonucleotides.

Supplementary Table S2. List of genes with altered expression in Δ*ter* strain of *Y. lipolytica* (see xls file in the Supplementary Dataset)

Supplementary Table S3: List of GO terms used for filtering of DEGs homologs with functions related to telomeres.

Supplementary Table S4. Nuclear genomes of the *Yarrowia* clade species deposited at the EMBL-ENA.

Captures of full-length gels and membranes used for preparation of Figures 2-5.

References

Court, R., Chapman, L., Fairall, L. & Rhodes, D. How the human telomeric proteins TRF1 and TRF2 recognize telomeric DNA: a view from high-resolution crystal structures. *EMBO Rep.* **6**, 39-45 (2005).

Brown, Y., Abraham, M., Pearl, S., Kabaha, M.M., Elboher & E., Tzfati, Y. A critical three-way junction is conserved in budding yeast and vertebrate telomerase RNAs. *Nucleic Acids Res.* **35**, 6280-6289 (2007).

Gunisova, S. *et al.* Identification and comparative analysis of telomerase RNAs from *Candida* species reveal conservation of functional elements. *RNA* **15**, 546–59 (2009).

Waldl, M. *et al.* TERribly difficult: searching for telomerase RNAs in Saccharomycetes. *Genes (Basel)* **9**, E372; 10.3390/genes9080372 (2018).

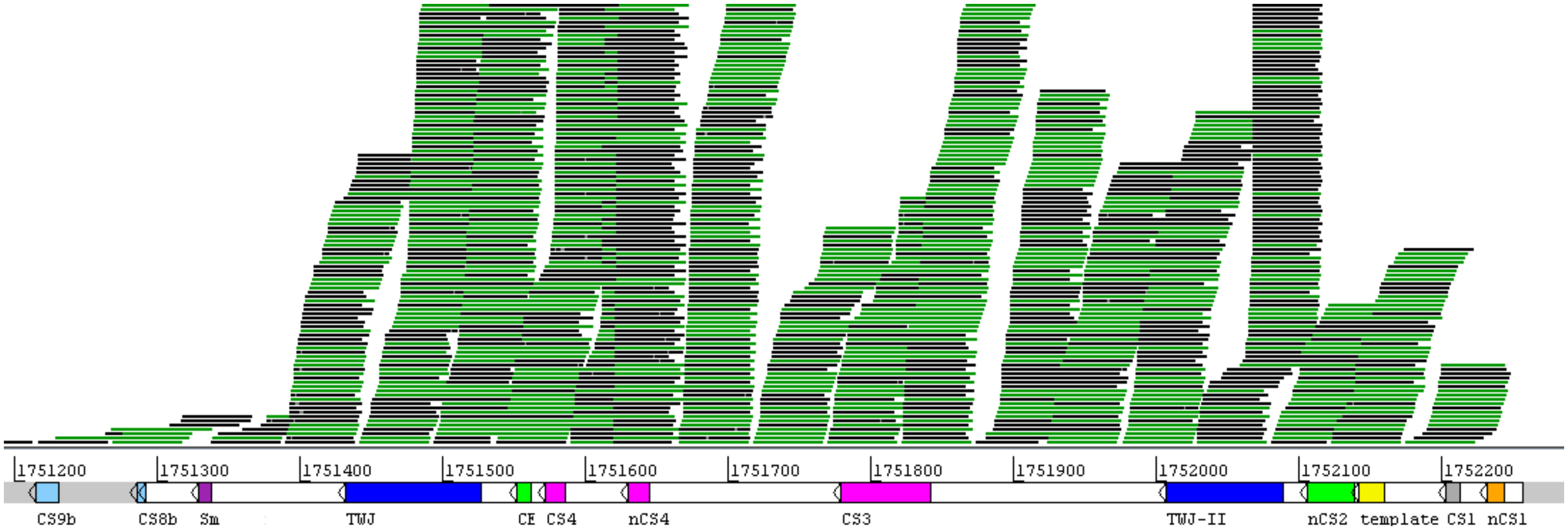

Supplementary Figure S1: Position of reads from RNA-seq analysis of the TER locus of *Y. lipolytica*. Ribo-depleted RNA were mapped to *Y. lipolytica* E150 reference genome and visualized with Artemis. Position of the main motifs are indicated by coloured rectangles. TWJ: three-way junction; Sm: Sm binding site; CS: conserved sequences.

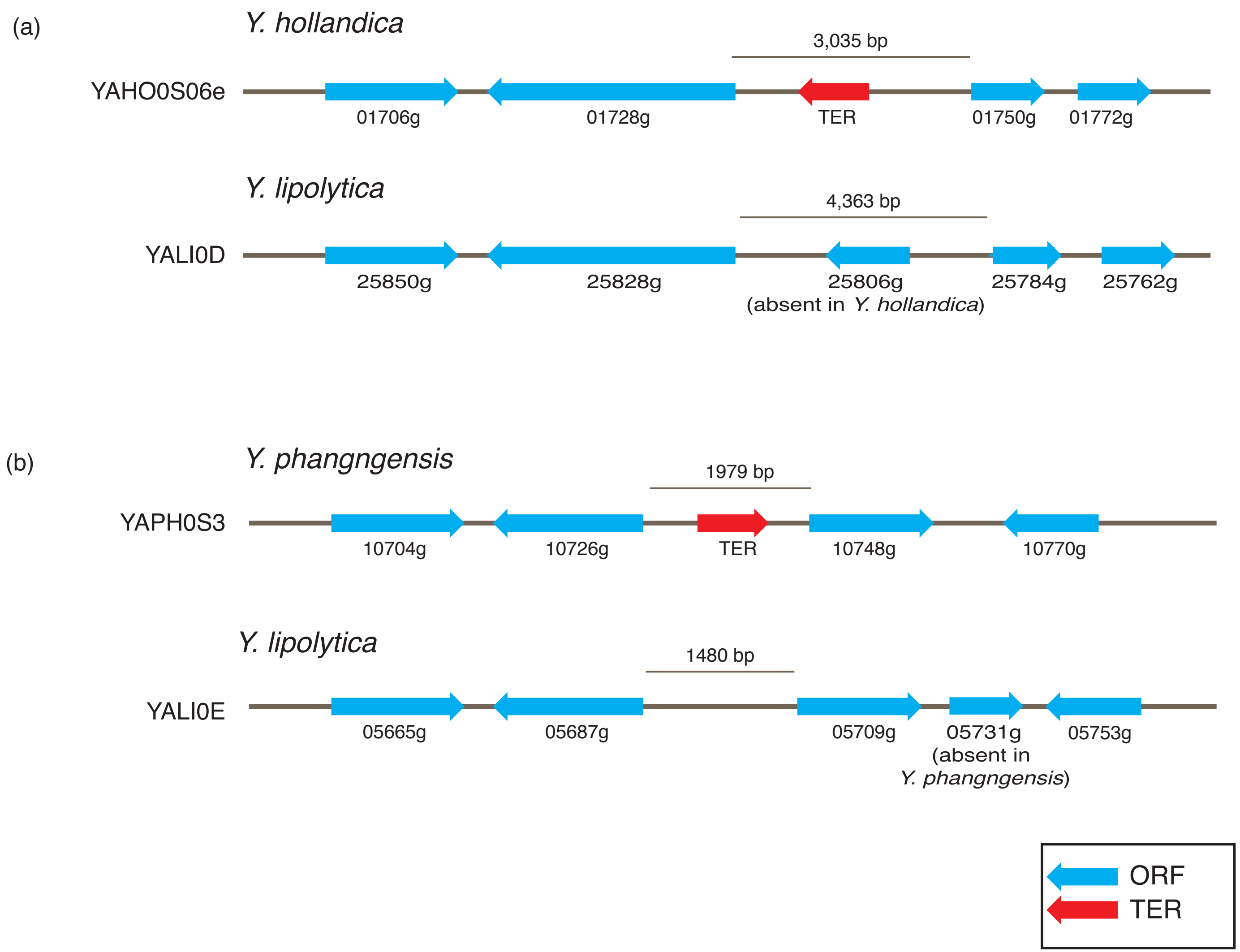

Supplementary Figure S2. Translocation events resulting in relocalization of TER locus in *Y. hollandica* (a) and *Y. phangngensis* (b).

```
                    Telomeric repeat                                        Myb1                                                            Myb2
                        (5'->3')   N-term                                                                     spacer                                                             C-term
Y. lipolytica     TTAGTCAGGG 1-150- VRLRWTQEETADLMEGCKVHGVGNWKKILTDPRFRFNNRTAVDLKDRFRTCFPE -205-237- ERRVFTPEEDERLLNGFMKHGP-SWSNIQRDNELGLFERRSTDLRDRFRNAFPL-291-406
Y. oslonensis     TTAGTCAGGG 1-150- VRLRWTQEETADLMEGCKVHGVGNWKKILTDPRFRFNNRTAVDLKDRFRTCFPE -205-237- ERRVFTPEEDERLLNGFMKHGP-SWSNIQRDNELGLFERRSTDLRDRFRNAFPL-291-406
Y. galli          TTAGTCAGGG 1-151- VRLRWTQEETADLMEGCKVHGVGNWKKILTDPRFRFNNRTAVDLKDRFRTCFPE -206-238- ERRVFTPEEDERLLNGFMKHGP-SWSNIQRDNELGLFERRSTDLRDRFRNAFPL-292-407
Y. divulgata      TTAGTCAGGG 1-151- VRLRWTQEETADLMEGCKVHGVGNWKKILTDPRFRFNNRTAVDLKDRFRTCFPE -206-238- ERRVFTPEEDERLLNGFMKHGP-SWSNIQRDNELGLFERRSTDLRDRFRNAFPL-292-407
Y. deformans      TTAGTCAGGG 1-151- VRLRWTQEETADLMEGCKVHGVGNWKKILTDPRFRFNNRTAVDLKDRFRTCFPE -206-238- ERRVFTPEEDERLLNGFMKHGP-SWSNIQRDNELGLFERRSTDLRDRFRNAFPL-292-405
Y. porcina        TTAGTCAGGG 1-152- VRLRWTQEESADLMEGCKVHGVGNWKKILTDPRFRFNNRTAVDLKDRFRTCFPE -207-239- ERRVFTPEEDERLLNGFMKHGP-SWSNIQRDSELGLFERRSTDLRDRFRNAFPV-293-404
Y. keelungensis   TTAGTCAGGG 1-152- VRLRWTQEETADLMEGCKVHGVGNWKKILTDPRFRFNNRTAVDLKDRFRTCFPE -206-238- ERRVFTPEEDERLLNGFMKHGP-SWSNIQRDNELGLFERRSTDLRDRFRNAFPL-292-405
Y. yakushimensis  TTATACAGGG 1-150- VRLRWTQEETADLMEGCKVHGVGNWKKILTDPRFRFNNRTAVDLKDRFRTCFPE -205-237- ERRVFTPEEDERLLNGFMKHGA-SWSNIQRDNELGLFERRSTDLRDRFRNAFPL-291-405
Y. bubula         TTAGCGAGGG 1-148- VRLRWTQEETADLMEGCKVHGVGNWKKILTDPRFRFNNRTAVDLKDRFRTCFPE -203-235- ERRVFTPEEDERLLNGFMKHGP-SWSNIQRDNELGLFERRSTDLRDRFRNAFPL-289-404
Y. hollandica     TTAGGCAGGG 1-149- VRLRWTQEETADLMEGCKVHGVGNWKKILTDPRFRFNNRTAVDLKDRFRTCFPE -204-236- ERRVFTPEEDERLLNGFMKHGP-SWSNIQRDNELGLFERRSTDLRDRFRNAFPL-289-404
Y. phangngensis   TTAGCCAGGG 1-158- VRLRWTQEETADLMEGCKVHGVGNWKKILTDPRFRFNNRTAVDLKDRFRTCFPE -213-245- ERRVFTPEEDERLLNGFMKHGP-SWSNIQRDNELGLFERRSTDLRDRFRNAFPL-299-411
Y. alimentaria    TTAGGCAGGG 1-149- VRLRWTQEETQDLMEGCKVHGVGNWKKILTDQRFRFNNRTAVDLKDRFRTCFPE -205-236- ERRVFTPEEDERLLNGFMKHGP-SWSNIQRDNDLGLFERRSTDLRDRFRNAFPL-291-406
C. hispaniensis   TTGACGAGAG 1-133- VRLRWTQEETSDLMEGCKQYGVGNWKKILTDPRFHFNNRTAVDLKDRFRTCFPE -188-221- ERRVFTPEEDERLLNGFMKHGP-SWSNIQRDHELGLYDRRSTDLRDRFRNAFPL-275-389

                                    -*--*--**---*--*-*-**-***--*-----*-*-*****-*******----           ***-*--**-*-*--*--***--***-*----------*-*--******----*
                        hTRF1-Myb   KRQAWLWEEDKNLRSGVRKYGEGNWSKILLH--YKFNNRTSVMLKDRWRTMKKL           KRQAWLWEEDKNLRSGVRKYGEGNWSKILLH--YKFNNRTSVMLKDRWRTMKKL
                        hTRF2-Myb   KKQKWTVEESEWVKAGVQKYGEGNWAAISKN--YPFVNRTAVMIKDRWRTMKRL           KKQKWTVEESEWVKAGVQKYGEGNWAAISKN--YPFVNRTAVMIKDRWRTMKRL
                                              helix 1            helix 2      helix 3                          helix 1            helix 2      helix 3
```

Supplementary Figure S3. Comparison of Myb domains of Tay1p homologs in the species belonging to the *Yarrowia* clade. Red letters indicate positions within telomeric repeats and Myb domains, respectively, that underwent substitutions compared with the corresponding sequences in *Y. lipolytica*. Shaded parts of the telomeric repeats indicate conserved positions. Both Myb domains are aligned to Myb domains of human TRF1 and TRF2 proteins. The regions corresponding to helices 1–3 are derived from Court et al. (2005). Asterisks indicate conserved positions.

Reference

Court, R., Chapman, L., Fairall, L. & Rhodes, D. How the human telomeric proteins TRF1 and TRF2 recognize telomeric DNA: a view from high-resolution crystal structures. *EMBO Rep*. 6, 39-45 (2005).

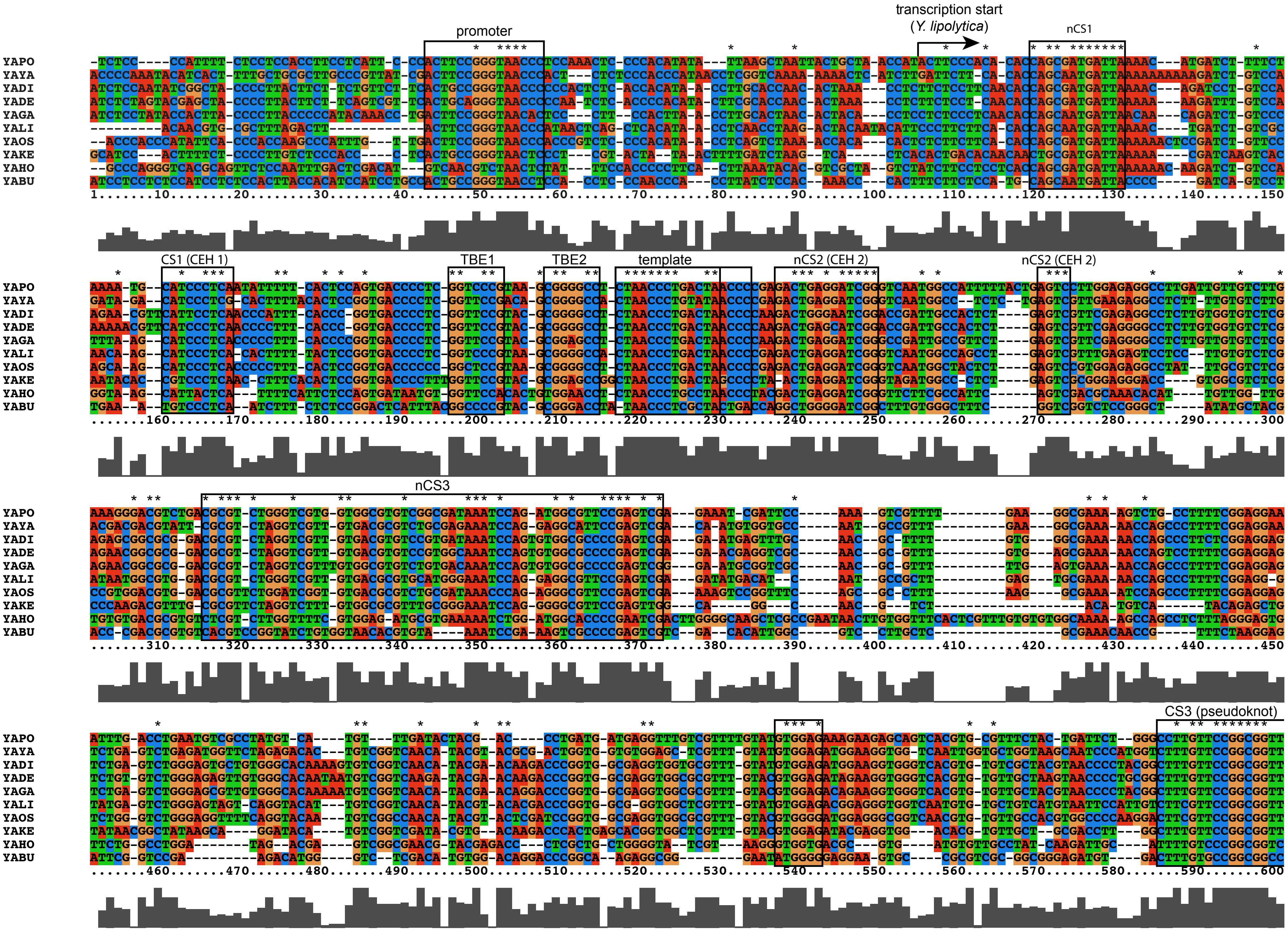

Supplementary Figure S4. Clustal X alignment of TER orthologs from the *Yarrowia* clade species.

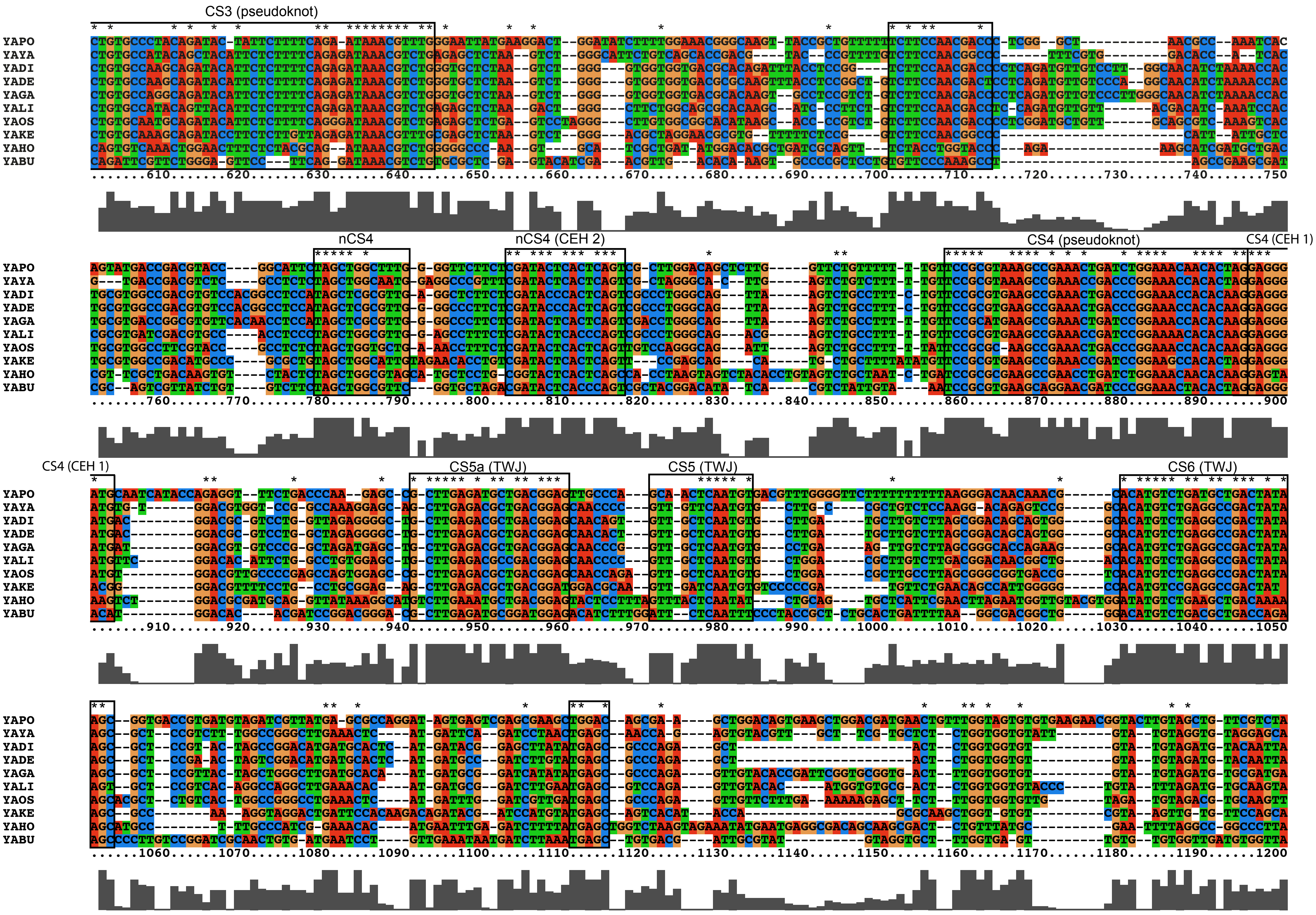

Supplementary Figure S4. *(continued)*

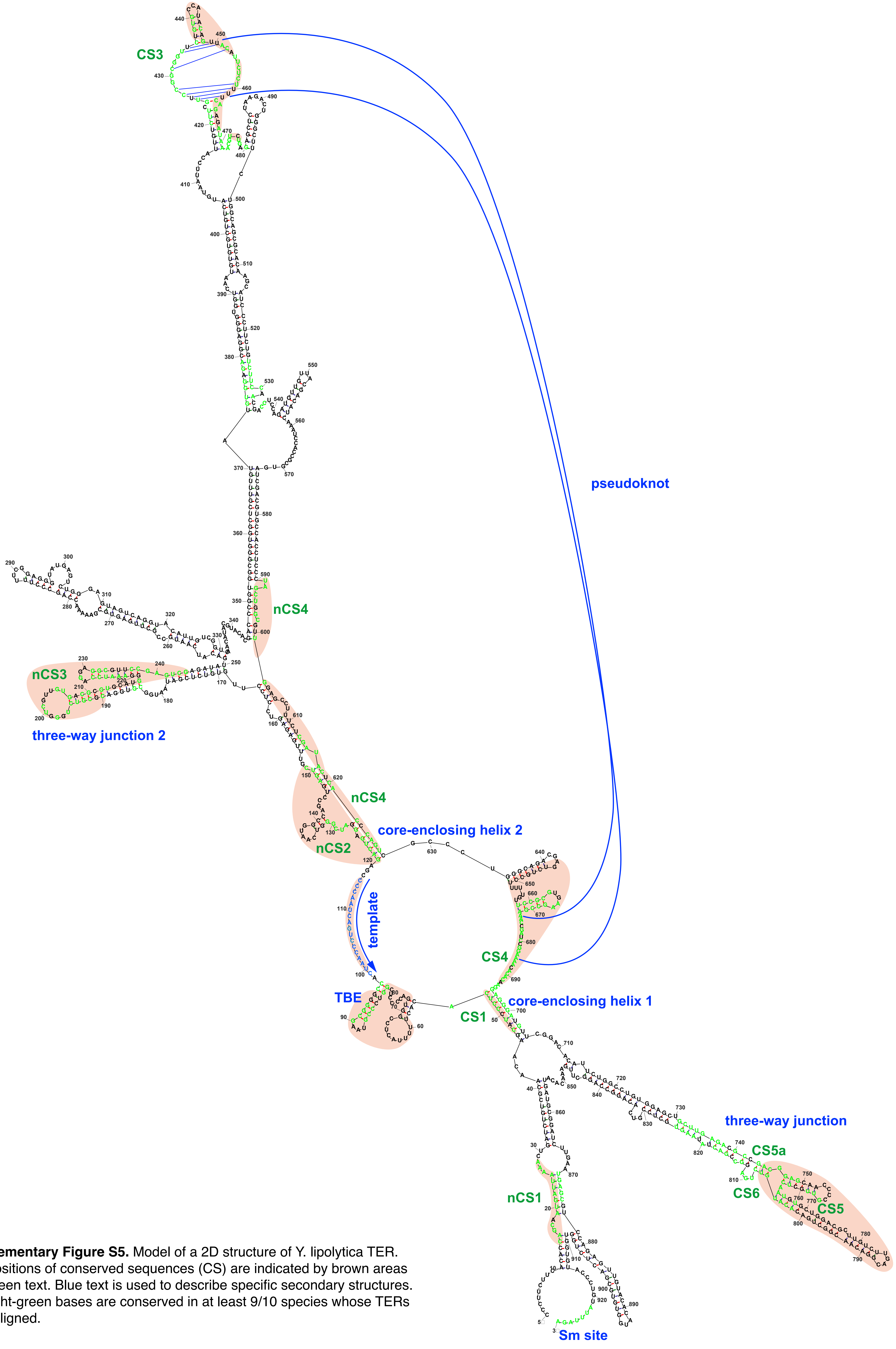

Supplementary Figure S5. Model of a 2D structure of Y. lipolytica TER. The positions of conserved sequences (CS) are indicated by brown areas and green text. Blue text is used to describe specific secondary structures. The light-green bases are conserved in at least 9/10 species whose TERs were aligned.

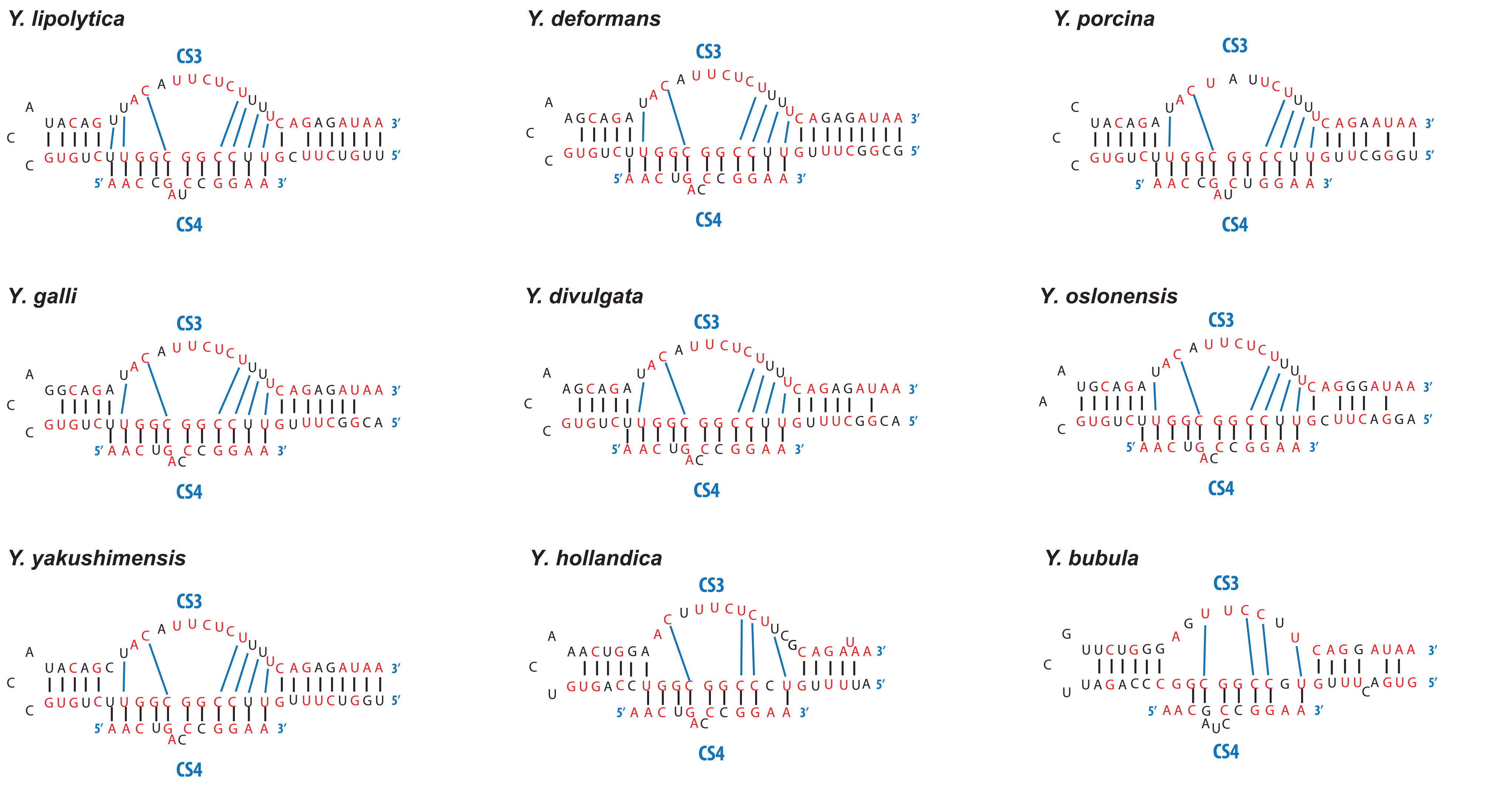

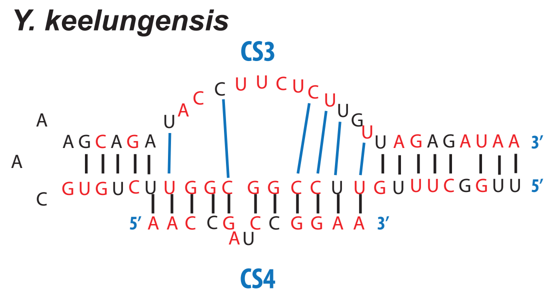

Supplementary Figure S6. Predicted structures of pseudoknots in TER from indicated *Yarrowia spp*. The two conserved sequences forming the pseudoknot (CS3, CS4) are indicated. The red bases are conserved in at least 9/10 species whose TERs were aligned.

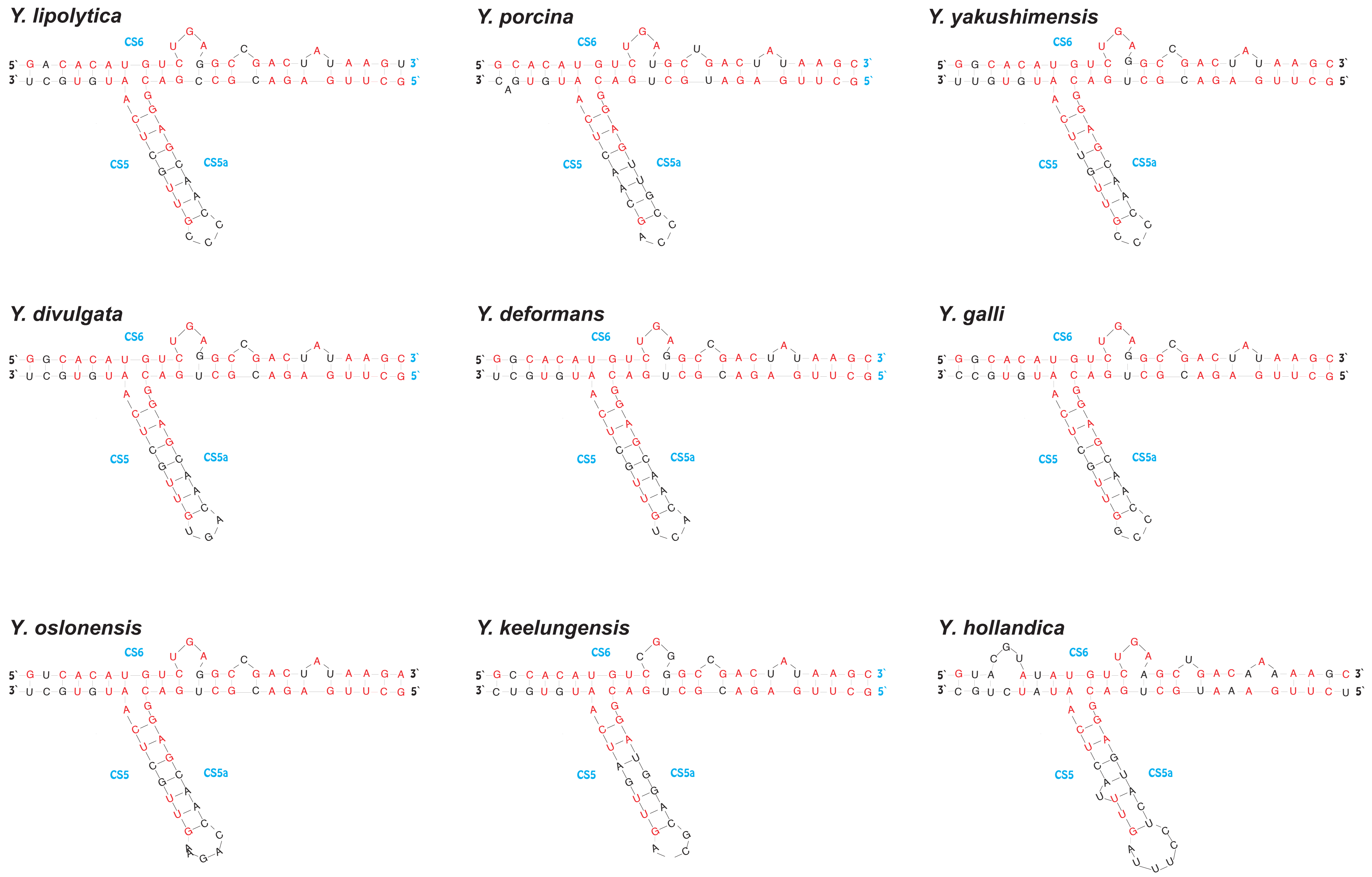

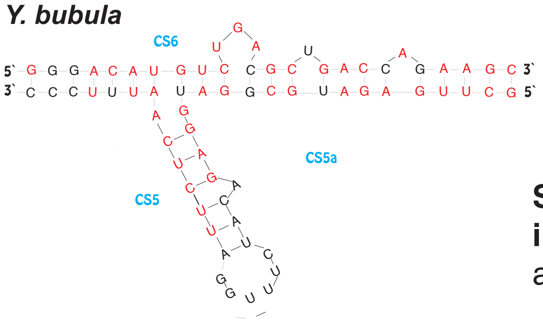

Supplementary Figure S7. Predicted structures of three-way junctions in TER from indicated *Yarrowia spp*. The three subunits of the three-way junction (CS5, CS5a, CS6) are indicated. The red bases are conserved in at least 9/10 species whose TERs were aligned.

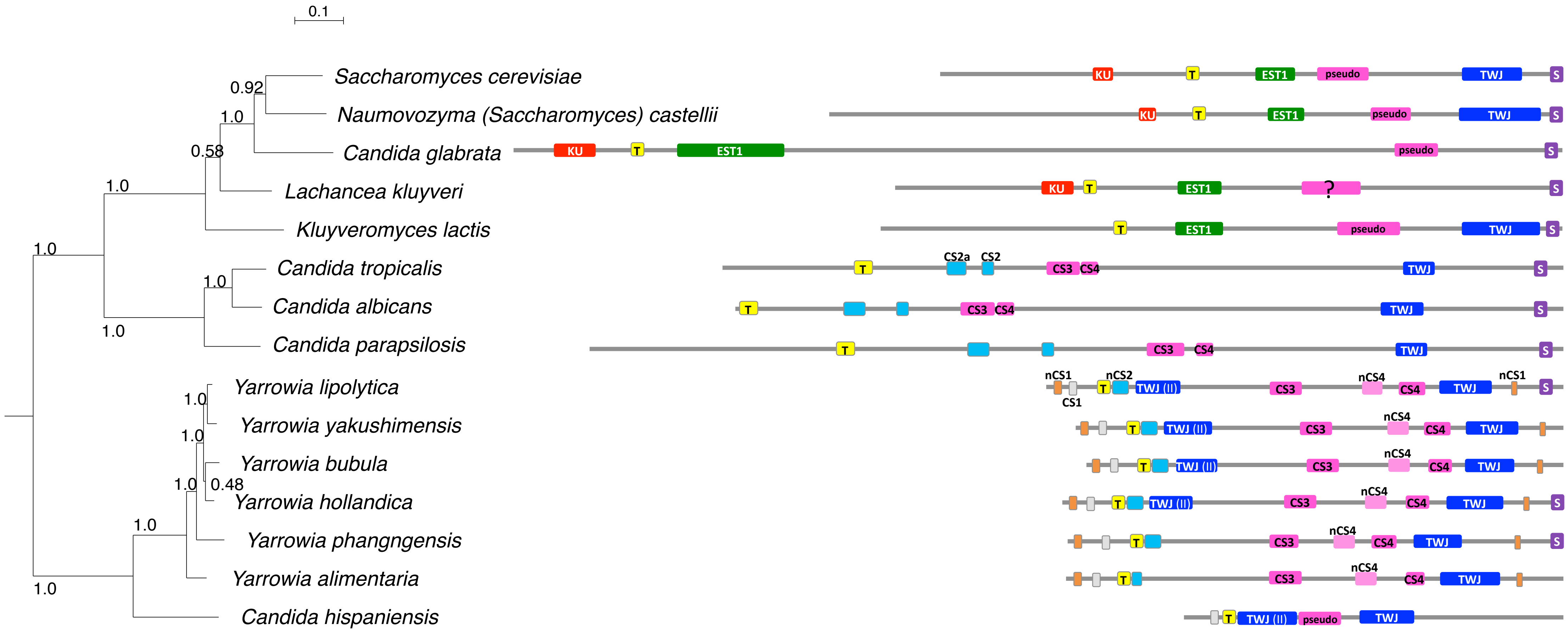

Supplementary Figure S8a. Main features and motifs in TER sequences of Saccharomycotina yeasts. The phylogenetic tree is based on the concatenation of 104 groups of orthologous proteins (40,077 residues) chosen with the criteria defined in Materials and methods. The tree was constructed with PhyML with a LG substitution model corrected by a Γ-law distribution, with four different categories of evolution rates. *Schizosaccharomyces pombe* was used as an outgroup. Branch support was estimated with aLRT non-parametric branch support based on a SH-like procedure, with seaview. Data for Saccharomycetaceae species are from Waldl et al. (2018), Kachouri-Lafond et al. (2009), and the Telomerase Database http://telomerase.asu.edu/; those for the *Candida albicans* clade from Gunisova et al. (2009). KU: ku binding hairpin; T: template region; EST1: Est1 binding site; TWJ: three-way junction; S: Sm binding site; CS1, nCS1, CS3, nCS4 and CS4 refer to conserved sequences; pseudo: pseudoknot including CS3 and CS4; ? putative pseudoknot with unresolved secondary structure.

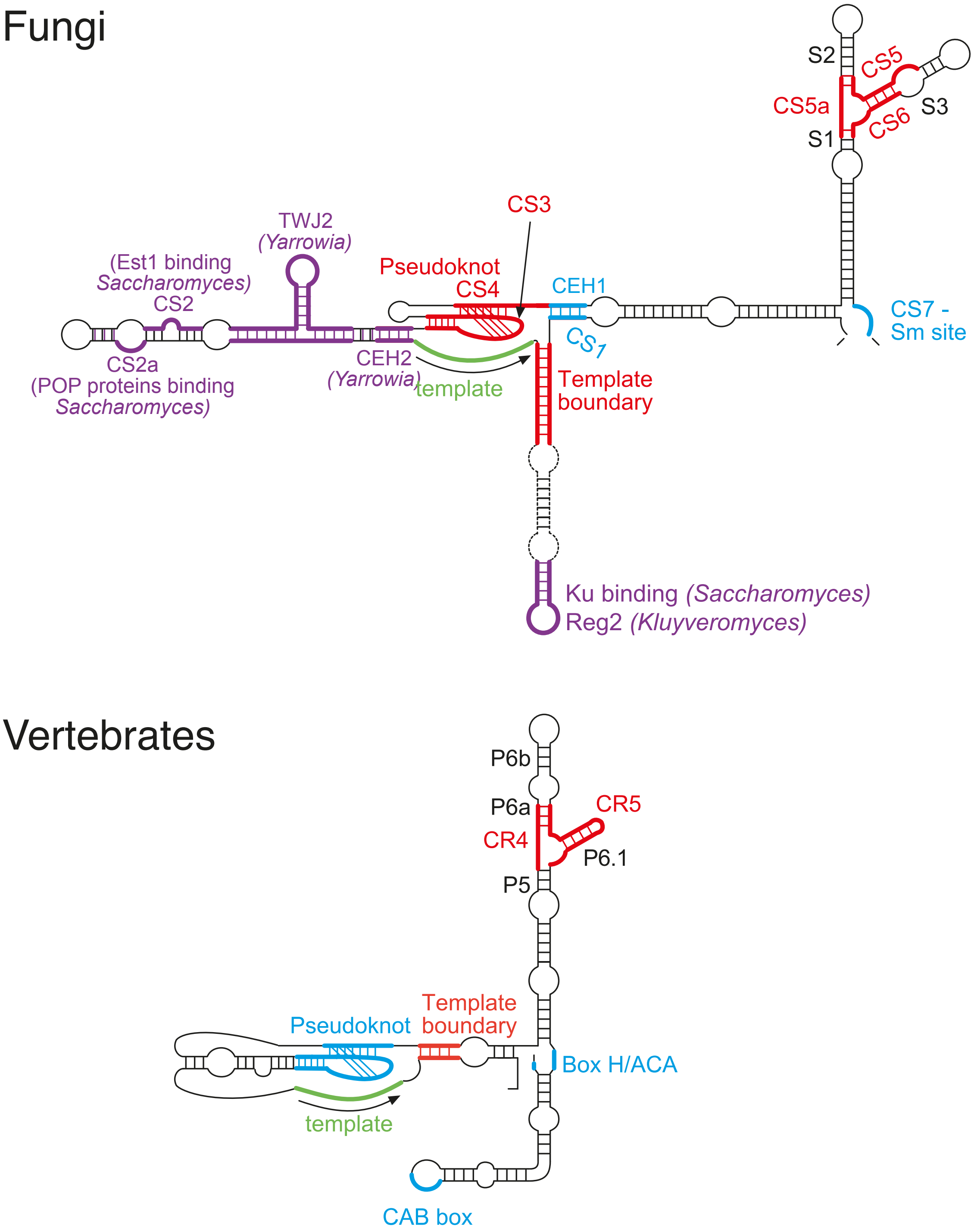

Supplementary Figure S8b. Schematic models for fungal and vertebrate TERs. In red, elements conserved across all fungal and vertebrate TERs examined. In blue, elements conserved among all fungal or all vertebrate TERs examined. In purple, elements conserved among some but not all groups of fungi. Based on Brown *et al.* (2007).

Supplementary Table S1. List of oligonucleotides (sequences are shown in 5'->3' direction).

Construction of the ΔTER strain

Amplification of TER locus	YITER_UP	TGTTCTTGGCGGATAGTAGAGGGAGGGCGA
Amplification of TER locus	YITER_DN	CGACCGAGTAGGCTCTGACAAATGGTGGCG
Amplification of URA3	URA3_5'_NdeI	AAACATATGCGGCCAGTGAATTGTAATACGACTCAC
Amplification of URA3	URA3_3'_BglII	AAAAGATCTGCTCGGAATTAACCCTCACTAAAGGG

Verification of the correct disruption of *Yl*TER gene

Amplificaiton of the 5' end	TER_5'_OP1	GCAGCACGGACTGTAAGAAGG
Amplificaiton of the 5' end	YIURA3_5'_antisense	TAGACTGGACTATACGGCTATCGG
Amplificaiton of the 3' end	TER_3'_OP1	CCGAAACGATGCTGCTATC
Amplificaiton of the 3' end	YIURA3_3'_sense	TCTCGCTAGGGATAACAGGGTAA

Ectopic expression of full-length *Yl*TER gene and the deletion variants

Expression of the full-length YITER	YITER_comp_UP	GCAAGCTTTTGTCTGTACCGAATAACATGATTGT
Expression of the full-length YITER	YITER_comp_DN	CGGGATCCTAGGAGCGGTGACAAAGTGAA
Deletion variant lacking template domain	tempTER_UP	GTGGCCCCGCTTAC
Deletion variant lacking template domain	tempTER_DN	CGAGACTGAGGATCGG
Deletion variant lacking template-boundary element	boundTER_UP	AGTGTGAGGGATGCT
Deletion variant lacking template-boundary element	boundTER_DN	CCACTAACCCTGACTAAC
Deletion variant lacking CS4 subunit of pseudoknot	knoTER_UP	CGTCTGCCCAGGG
Deletion variant lacking CS4 subunit of pseudoknot	knoTER_DN	AGGAGGGATGTTCGG
Deletion variant lacking CS3 subunit of pseudoknot	estTER_UP	CGAAGACAATGGAATTACATGACA
Deletion variant lacking CS3 subunit of pseudoknot	estTER_DN	AGAGCTCTAAGACTGGGCTTCTG
Deletion variant lacking motif 1	dom1TER_UP	GAACCGCCGGAACG
Deletion variant lacking motif 1	dom1TER_DN	TTCTCTTTTCAGAGATAAACGTCTGA
Deletion variant lacking motif 2	dom2TER_UP	ATGTAACTGTATGGCACAGA
Deletion variant lacking motif 2	dom2TER_DN	TAAACGTCTGAGAGCTCTAAG
Deletion variant lacking motif 3	dom3TER_UP	GCTCTCAGACGTTTATCTCTGA
Deletion variant lacking motif 3	dom3TER_DN	AAGACTGGGCTTCTGGC
Deletion variant lacking core-enclosing helix	coreTER_UP	CTTGTGTGTTTTCCGGAT
Deletion variant lacking core-enclosing helix	coreTER_DN	TCGGACACATTCTGGC
Deletion variant lacking three-way junction	csdelTER_UP	CGGCGTCTCAAGCAGCTC
Deletion variant lacking three-way junction	csdelTER_DN	TGAGGCCGACTATAAGTCCTCC
Deletion variant lacking Sm site	nsmTER_UP	AGGGTACACCACCAGAGT
Deletion variant lacking Sm site	nsmTER_DN	TGTGCAAGTAGACCCACTG
Deletion variant lacking nCS1	ele1TER_UP	AAACTGATCTGTCGCA

Deletion variant lacking nCS1	ele1TER_DN	GTGTGAAGAAGGGAATG
Deletion variant lacking nCS2	ele2TER_UP	GTTTGAGAGTCCTCCTT
Deletion variant lacking nCS2	ele2TER_DN	TCGGGGTTAGTCAG
Deletion variant lacking nCS3	CS1nTER_UP	GATATGACATCAATGCC
Deletion variant lacking nCS3	CS1nTER_DN	TCCACGCCATTATC
Deletion variant lacking nCS4	NCS2nTER_UP	GGGAGGTGGCACGTCG
Deletion variant lacking nCS4	NCS2nTER_DN	GCCCTGGGCAGACGAG
Deletion variant lacking promoter	promTER_UP	ATAACTCAGCTCACATAACC
Deletion variant lacking promoter	promTER_DN	AAGTCTAAAGCGCACG

Probes for EMSA, In-gel hybridization and TRF analysis

Probe representing 2 telomeric repeats of *Y. lipolytica*	YlipTEL_G	TTAGTCAGGGTTAGTCAGGG
Probe representing 2 telomeric repeats of *Y. lipolytica*	YlipTEL_C	CCCTGACTAACCCTGACTAA
Probe representing 2 telomeric repeats of *Y. alimentaria*	CaliTEL_G	TTAGGCAGGGTTAGGCAGGG
Probe representing 2 telomeric repeats of *Y. alimentaria*	CaliTEL_C	CCCTGCCTAACCCTGCCTAA
Probe representing 2 telomeric repeats of *Y. phangngensis*	CphaTEL_G	TTAGCCAGGGTTAGCCAGGG
Probe representing 2 telomeric repeats of *Y. phangngensis*	CphaTEL_C	CCCTGGCTAACCCTGGCTAA
Probe representing 2 telomeric repeats of *Y. bubula*	YbubTEL_G	TTAGCGAGGGTTAGCGAGGG
Probe representing 2 telomeric repeats of *Y. bubula*	YbubTEL_C	CCCTCGCTAACCCTCGCTAA
Probe representing 2 telomeric repeats of *Y. yakushimensis*	YyakTEL_G	TTATACAGGGTTATACAGGG
Probe representing 2 telomeric repeats of *Y. yakushimensis*	YyakTEL_C	CCCTGTATAACCCTGTATAA
Probe representing 2 telomeric repeats of *C. hispaniensis*	ChisTEL_G	TTGACGAGAGTTGACGAGAG
Probe representing 2 telomeric repeats of *C. hispaniensis*	ChisTEL_C	CTCTCGTCAACTCTCGTCAA
Probe for the In-gel hybridization	YITEL probe 2	CCCTGACTAACCCTGACTAA
Probe for the TRF analysis	YITEL probe 1	$(TTAGTCAGGG)_{81}$

Supplementary Table S3: List of GO terms used for filtering of DEGs' homologs with functions related to telomeres.

GO number	Category description
GO:0000723	telomere maintenance
GO:0000781	chromosome telomeric region
GO:0000783	nuclear telomere cap complex
GO:0000784	nuclear chromosome telomeric region
GO:0001309	age-dependent telomere shortening
GO:0003691	double-stranded telomeric DNA binding
GO:0007004	telomere maintenance via telomerase
GO:0010521	telomerase inhibitor activity
GO:0010833	telomere maintenance via telomere lengthening
GO:0010834	telomere maintenance via telomere shortening
GO:0016233	telomere capping
GO:0031627	telomeric loop formation
GO:0031848	protection from non-homologous end joining at telomere
GO:0032203	telomere formation via telomerase
GO:0032205	negative regulation of telomere maintenance
GO:0032206	positive regulation of telomere maintenance
GO:0032211	negative regulation of telomere maintenance via telomerase
GO:0032212	negative regulation of telomere maintenance via telomerase
GO:0032214	negative regulation of telomere maintenance via semi-conservative replication
GO:0042162	telomeric DNA binding
GO:0043047	single-stranded telomeric DNA binding
GO:0051973	positive regulation of telomerase activity
GO:0051974	negative regulation of telomerase activity
GO:0070188	Stn1-Ten1 complex
GO:0070198	protein localization to chromosome telomeric region
GO:0098505	G-rich strand telomeric DNA binding

Supplementary Table S4. Nuclear genomes of *Yarrowia* species deposited at the EMBL-ENA

Species	Strain	Accession number	scaffolds	Assembly (bp)
Y. alimentaria	CBS 10151	PRJEB18080	11	19,830,970
Y. bubula	CBS 12934	PRJEB18081	29	20,901,952
Y. deformans	CBS 2071	PRJEB18082	42	21,059,484
Y. divulgata	CBS 11013	PRJEB18083	19	21,242,122
Y. galli	CBS 9722	PRJEB18084	6	22,823,274
Y. hollandica	CBS 4855	PRJEB18085	18	20,021,922
Y. keelungensis	CBS 11062	PRJEB18086	39	21,639,051
Y. oslonensis	CBS 10146	PRJEB18087	37	23,209,503
Y. phangngaensis	CBS 10407	PRJEB18088	9	16,207,847
Y. porcina	CBS 12935	PRJEB18089	43	30,708,733
Y. yakushimensis	CBS 10253	PRJEB18090	7	18,838,649
C. hispaniensis	CBS 9996	PRJEB18079	6	10,652,573

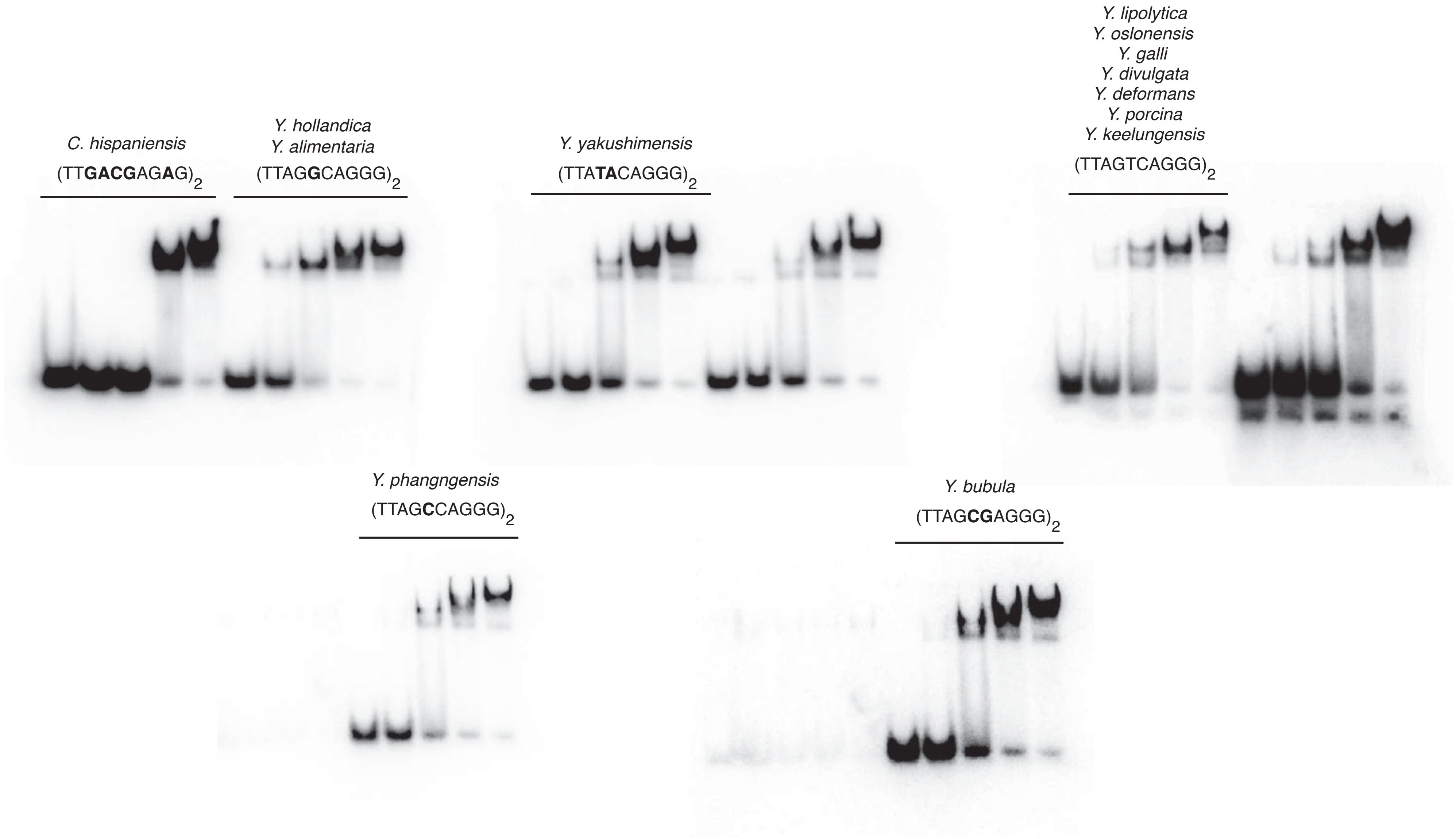

Full length gels for Figure 2.

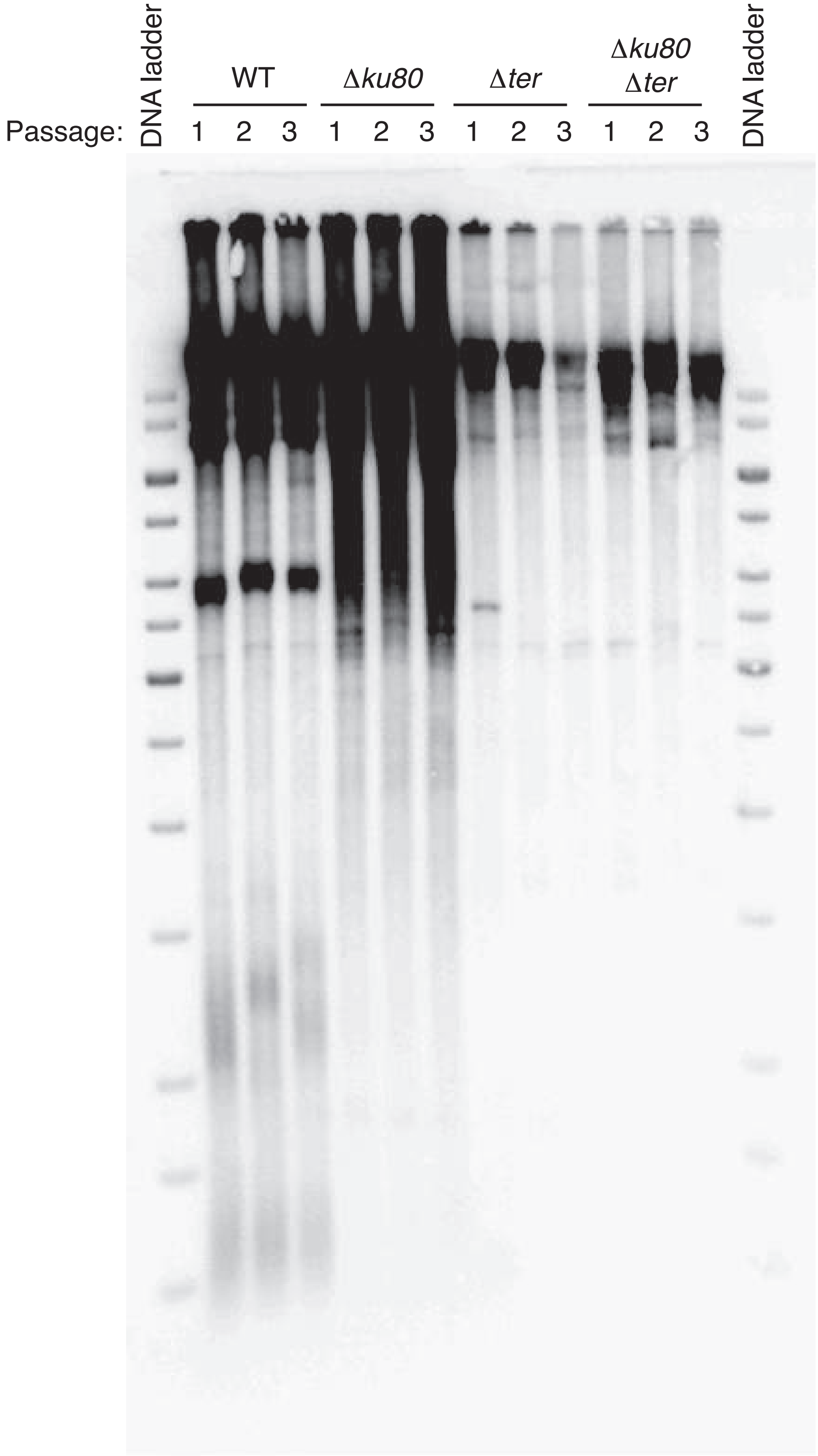

Full-length gels for Figure 3a.

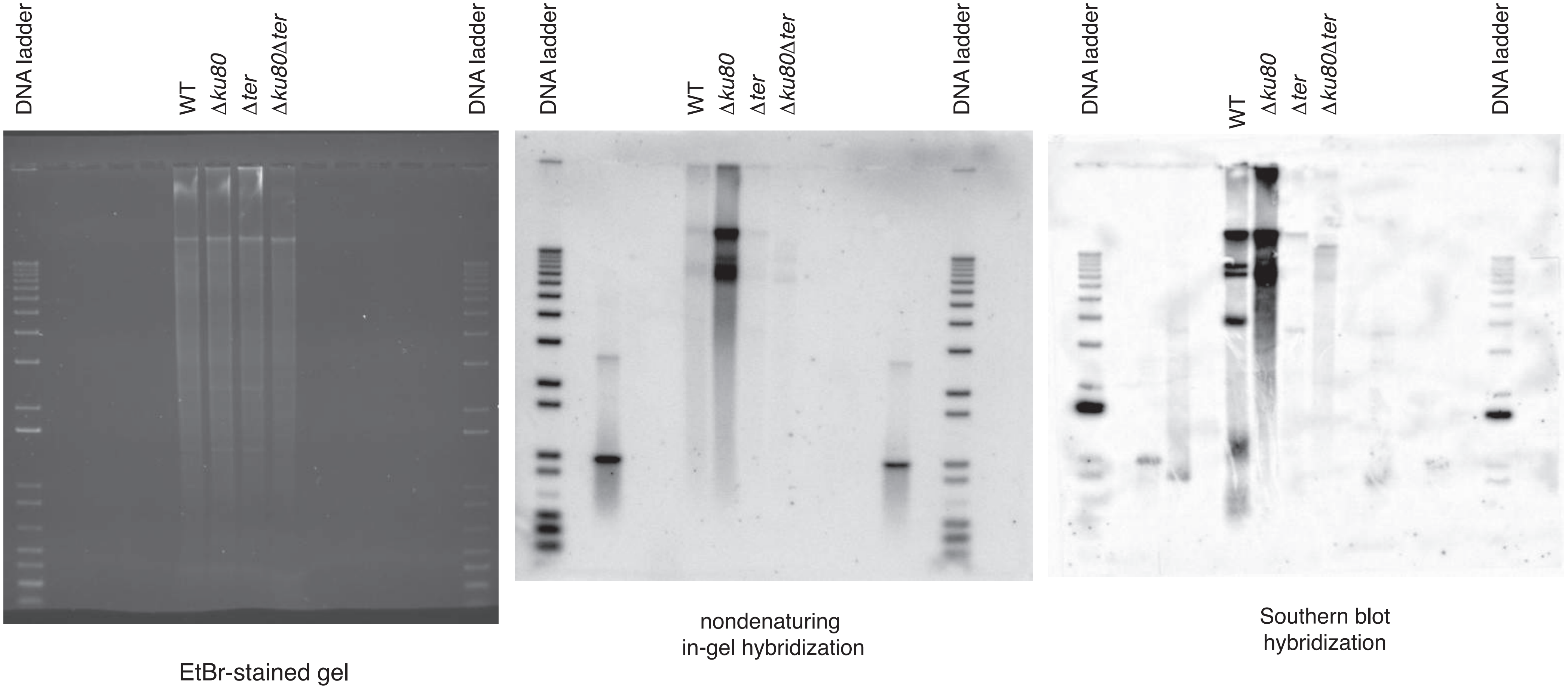

Full-length gels for Figure 3c.

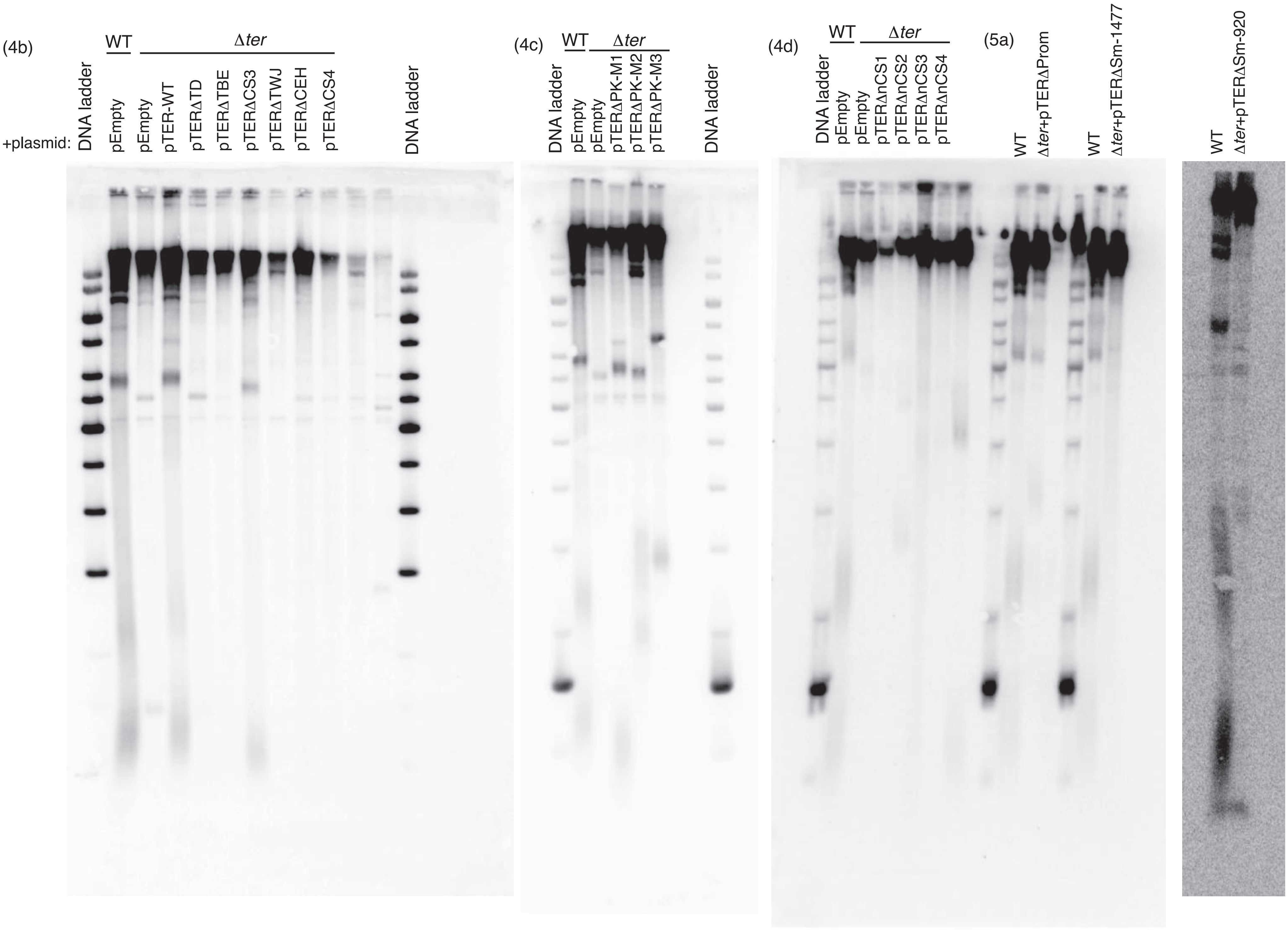

Full-length gels for Figure 4+5
